# Biochemical and structural insights into Rab12 interactions with RILP and its family members

**DOI:** 10.1038/s41598-021-89394-y

**Published:** 2021-05-13

**Authors:** Jana Omar, Efrat Rosenbaum, Adi Efergan, Bayan Abu Sneineh, Adva Yeheskel, Yuto Maruta, Mitsunori Fukuda, Ronit Sagi-Eisenberg

**Affiliations:** 1 Department of Cell and Developmental Biology, Sackler Faculty of Medicine, Tel Aviv University, 69978 Tel Aviv, Israel; 2 Bioinformatics Unit, Faculty of Life Sciences and Computational assisted drug-design unit, Blavatnik Center for Drug Discovery, Tel-Aviv University, 69978 Tel Aviv, Israel; 3 Laboratory of Membrane Trafficking Mechanisms, Department of Integrative Life Sciences, Graduate School of Life Sciences, Tohoku University, Aoba-ku, AobayamaSendai, Miyagi 980-8578 Japan

**Keywords:** Cell biology, Computational biology and bioinformatics, Immunology

## Abstract

Alongside its biosynthetic functions, the small GTPase Rab12 negatively regulates mast cell (MC) exocytosis by its interaction with RILP to promote retrograde transport of the MC secretory granules. Given the role of Rab effectors in mediating Rab functions, in this study we used biochemical and in silico tools to decipher Rab12 interactions with its RILP family effectors. We show that Rab12 interacts with RILP, RILP-L1 and RILP-L2 independently of each other, whereby lysine-71, in mouse Rab12, is critical for Rab12 interactions with RILP-L1 or RILP-L2, but is dispensable for the binding of RILP. Focusing on RILP, and relying on molecular dynamics simulations, functional mutational analyses and peptide inhibition assays, we propose a model for the Rab12-RILP complex, consisting of a RILP homodimer and a single molecule of active Rab12, that interacts with the RILP homology domain (RHD) of one RILP monomer and a C-terminal threonine in the other monomer via its switch I and switch II regions. Mutational analyses of RILP RHD also demonstrate its involvement in the regulation of MC secretory granule transport. Jointly, our results provide structural and functional insights into the Rab12-RILP complex on the basis of which new tools could be generated for decoding Rab12 functions.

## Introduction

A screen of Rab GTPases for their functional and phenotypic impact on mast cell (MC) exocytosis has identified 30 Rabs as potential regulators of this process^1^. Among these Rabs, a constitutively active mutant of Rab12 was found to inhibit exocytosis by stimulating microtubule dependent retrograde transport of the MC secretory granules (SGs), promoting their perinuclear clustering^1,2^. Rab12 is one of the less characterized Rabs. Our previous studies have implicated Rab12 in controlling transport of specific cargo, such as the transferrin receptor, from the endocytic recycling compartment (ERC) to lysosomes^3^ and stimulating autophagy by regulating the transport of the amino acid transporter PAT4^4^. Further studies implicated Rab12 in autophagosome trafficking^5^ and retrograde transport of the shiga toxin^6^. However, the underlying mechanisms of the diverse functions of Rab12 remain poorly understood. Rab GTPases perform their functions by the recruitment of effector proteins that bind to their active, GTP-bound conformation. The latter include motor proteins, SNAREs, tethering factors, cytoskeleton and cargo proteins, whose recruitment allow Rabs to regulate distinct steps along vesicular trafficking^7^. We identified the RILP homolog RILP-like 1 (RILP-L1) as an effector of Rab12 by yeast two hybrid screening^8^. We further confirmed the assignment of RILP-L1 as a Rab12 effector by pulldown assays, and also demonstrated the ability of Rab12 to pull down RILP, in agreement with RILP involvement in mediating Rab12-regulated minus end transport of the MC SGs^2^. Recent studies have demonstrated the association of Rab12 with RILP-like 2 (RILP-L2), another member of the RILP family^9^. Collectively, these results demonstrate that Rab12 binds all three members of the RILP family. Therefore, Rab12 overall functionality is likely to be dictated by its balanced connectivity. Focusing on Rab12 interaction with RILP, here we combined in silico modelling with mutational and functional analyses to gain structural insights into the Rab12-RILP complex and identify critical residues that mediate interactions within this complex.

## Results

### The RILP family members, RILP, RILP-L1 and RILP-L2, form homodimers, but do not heterodimerize with each other

**Figure 1:**
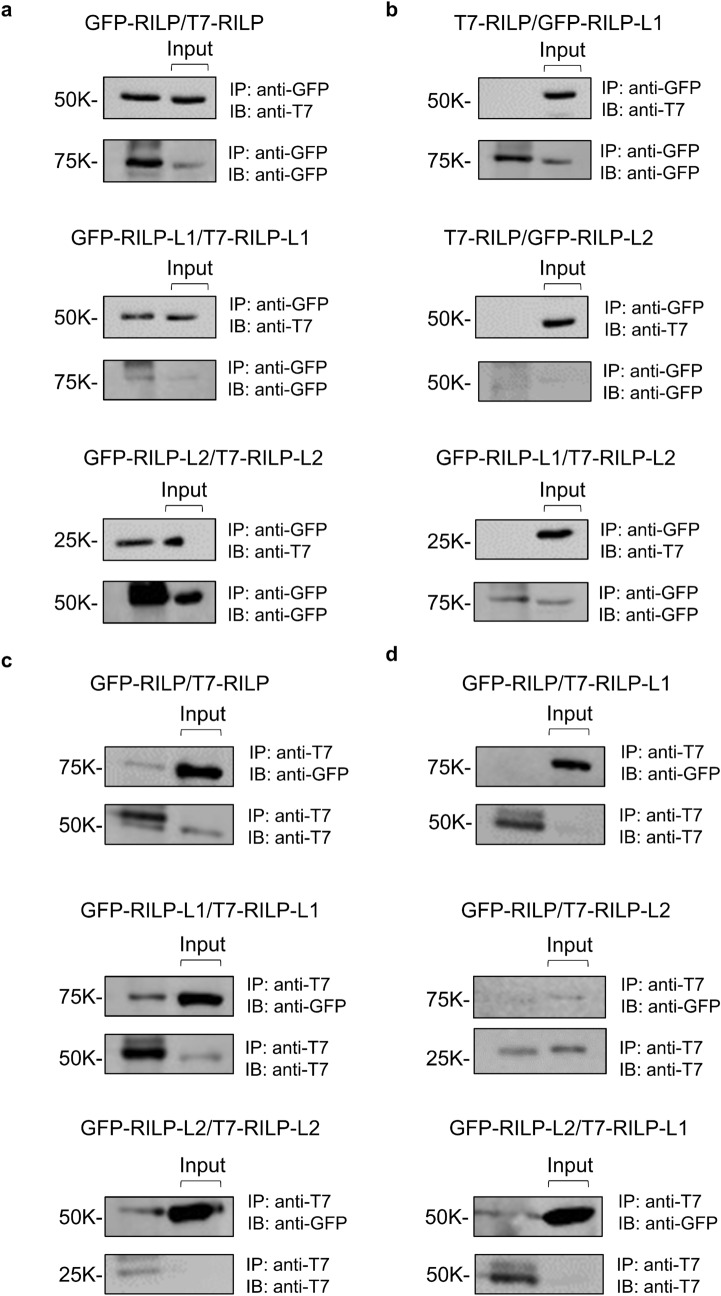
RILP, RILP-L1 and RILP-L2 may form homocomplexes, but neither protein can form heterocomplexes. RBL cell lysates derived from cells co-transfected with 17.5 μg of pEGFP plasmid encoding either RILP, RILP-L1 or RILP-L2, and 17.5 μg of pEF-BOS plasmid encoding either T7-RILP, T7-RILP-L1 or T7-RILP-L2, as indicated, were subjected to immunoprecipitation with rabbit polyclonal antibodies directed against GFP (**a**,**b**) or mouse monoclonal antibodies directed against the T7 epitope (**c**,**d**). Immune complexes were then analyzed by SDS-PAGE and immunoblotting with either mouse monoclonal anti T7 antibodies, followed by reprobing with polyclonal anti GFP antibodies (**a**,**b**), or polyclonal anti GFP antibodies, followed by reprobing with monoclonal anti T7 antibodies (**c**,**d**). Input = 10% of total protein.

Though Rab12 was shown to interact with its RILP family members in pulldown assays^2^, yeast two hybrid screening identified only Rab12 binding to RILP-L1^8^. This discrepancy prompted us to explore the possibility that RILP or RILP-L2 may interact with Rab12 by forming a heterodimer with RILP-L1. We relied on the observation that RILP was shown to form homodimers^10^, and therefore asked whether the other members of the family can form homo- or heterodimers. To this purpose, we examined the capacity of GFP-fused versions of RILP, RILP-L1 and RILP-L2 to co-immunoprecipitate T7-tagged versions of themselves or the other members of this family, which we co-transfected in RBL cells, our model MCs^2^. The results of these experiments confirmed the ability of RILP to form homodimers or homo complexes (Fig. 1a). Further, they also demonstrated the ability of GFP-RILP-L1 to co-immunoprecipitate T7-RILP-L1, and that of GFP-RILP-L2 to co-immunoprecipitate T7-RILP-L2 (Fig. 1a). Therefore, our results demonstrate that similarly to RILP, also the two other members of this family, RILP-L1 and RILP-L2, share the capacity of homodimerization. In sharp contrast, none of the RILP family members was able to co-immunoprecipitate any of the other members of this family. Hence, immunoprecipitated GFP-RILP failed to co-immunoprecipitate T7-RILP-L1 or T7-RILP-L2, and neither did GFP-RILP-L1 co-immunoprecipitate with T7-RILP-L2 (Fig. 1b). Similar results were obtained in reciprocal experiments, where the ability of T7-tagged RILP, RILP-L1 or RILP-L2, to co-immunoprecipitate their co-transfected, GFP-fused versions, was tested (Fig. 1c,d). Interestingly, testing the interactions between RILP members by two additional methods, the yeast two hybrid system and pulldown assays, clearly detected the homodimerization of RILP or RILP-L2 (Supplementary figures S1 and S2), however, RILP-L1 showed a lower capacity to homodimerize in the yeast two hybrid system (Supplementary figures S1), while no homodimerization was detected in the pulldown assays, where GST-RILP-L1 was unable to pull down GFP-RILP-L1 from RBL cell lysates (Supplementary figure S2). Notably, under similar experimental conditions, GST-RILP-L1 has effectively pulled down GTPγS-loaded Rab12 from the RBL cell lysates (Supplementary figure S2). Therefore, the lack of pulldown by GST-RILP-L1 was not due to malfunction of the fusion protein. Whether RILP-L1 undergoes in intact cells a posttranslational modification that facilitates its homodimerization in co-immunoprecipitation assays, unlike its interactions in the yeast two hybrid or pulldown assays, is presently unknown. However, since in neither method did any RILP member demonstrate heterodimerization activity (Fig. 1 and Supplementary figures S1 and S2), these results indicate that their interactions with Rab12 are independent of each other.

### Lysine 71 is critical for Rab12 binding of RILP-L1 and RILP-L2, but is dispensable for binding of RILP

**Figure 2:**
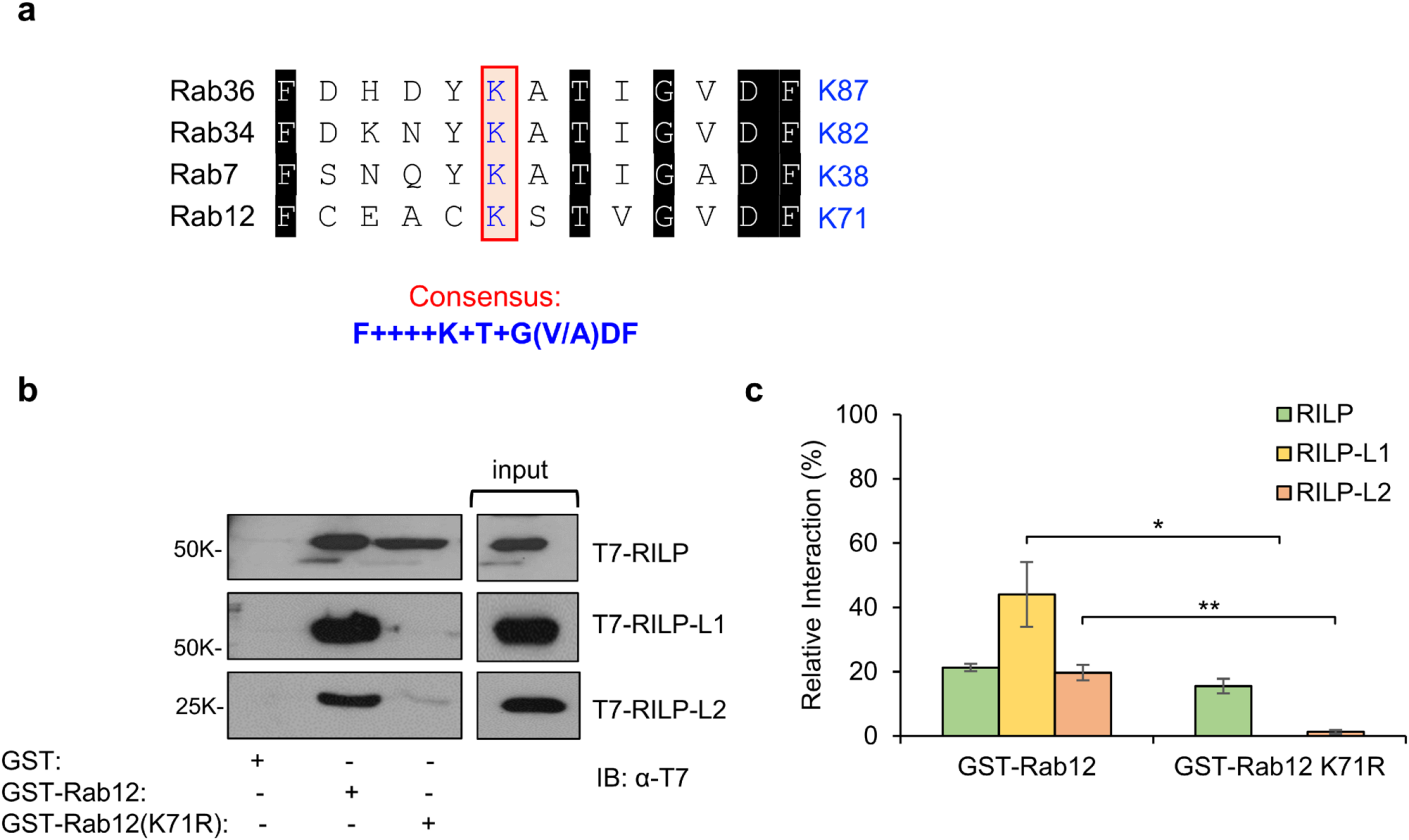
Delineating Rab12 binding sites for RILP family effectors. (**a**) Proposed consensus sequence based on sequence similarity of the regions neighbouring the lysine residues that are important for RILP binding to Rab7 and Rab34 (boxed). (**b**) Cell lysates (500 μg) derived from RBL cells that were transiently transfected with 35 μg of either pEF-T7-RILP, pEF-T7-RILP-L1, or pEF-T7-RILP-L2, were incubated for 18 h at 4 °C with 20 μg of GST, or GST-Rab12 or GST-Rab12(K71R), immobilized on glutathione agarose beads, in the presence of 0.5 mM GTPγS. Bound proteins were resolved by SDS-PAGE and analysed by immunoblotting with anti-T7 antibodies. Input = 10% of total protein. A representative blot is shown. (**c**) Blots were quantified by the ImageJ software and binding is presented as % of total input. Results are the average ± SEM derived from three independent experiments. *P[RILP-L1: GST-Rab12/GST-Rab12(K71R)] = 0.014, **P[(RILP-L2: GST-Rab12/GST-Rab12(K71R)] = 0.0013.

Aiming to delineate the binding site of Rab12 for its RILP family effectors, we relied on studies, which identified lysine 38 and 82 in Rab7 and Rab34, as critical for their interactions with RILP^10,11^. Sequence alignment of the amino acids that are proximal to those lysine residues, prompted us to propose the amino acid sequence F++++K+T+G(V/A)DF, that is also present in Rab36, another RILP-interacting protein^12^, and in Rab12 (Fig. 2a), as a consensus for RILP binding. Therefore, we substituted lysine 71, the corresponding lysine in Rab12, to arginine, that preserves the positive charge of the amino acid, but may interfere with its specific functions, and examined the impact of this mutation on Rab12 pulldown efficacy. In contrast to our expectation, GST-Rab12(K71R) retained its capacity to pull down T7-tagged RILP from RBL cell lysates (Fig. 2b,c). However, this mutation significantly inhibited the ability of Rab12 to pull down either RILP-L1 or RILP-L2 (Fig. 2b,c). Therefore, while these results support the positioning of K-71 at Rab12 binding site of RILP-L1 and RILP-L2, they imply that Rab12 binding site of RILP might either be distinct or redundant.

### Molecular dynamics simulations of the Rab12-RILP complex predict a ternary complex that involves two interfaces within Rab12 and the RILP Homology Domain (RHD)

**Figure 3:**
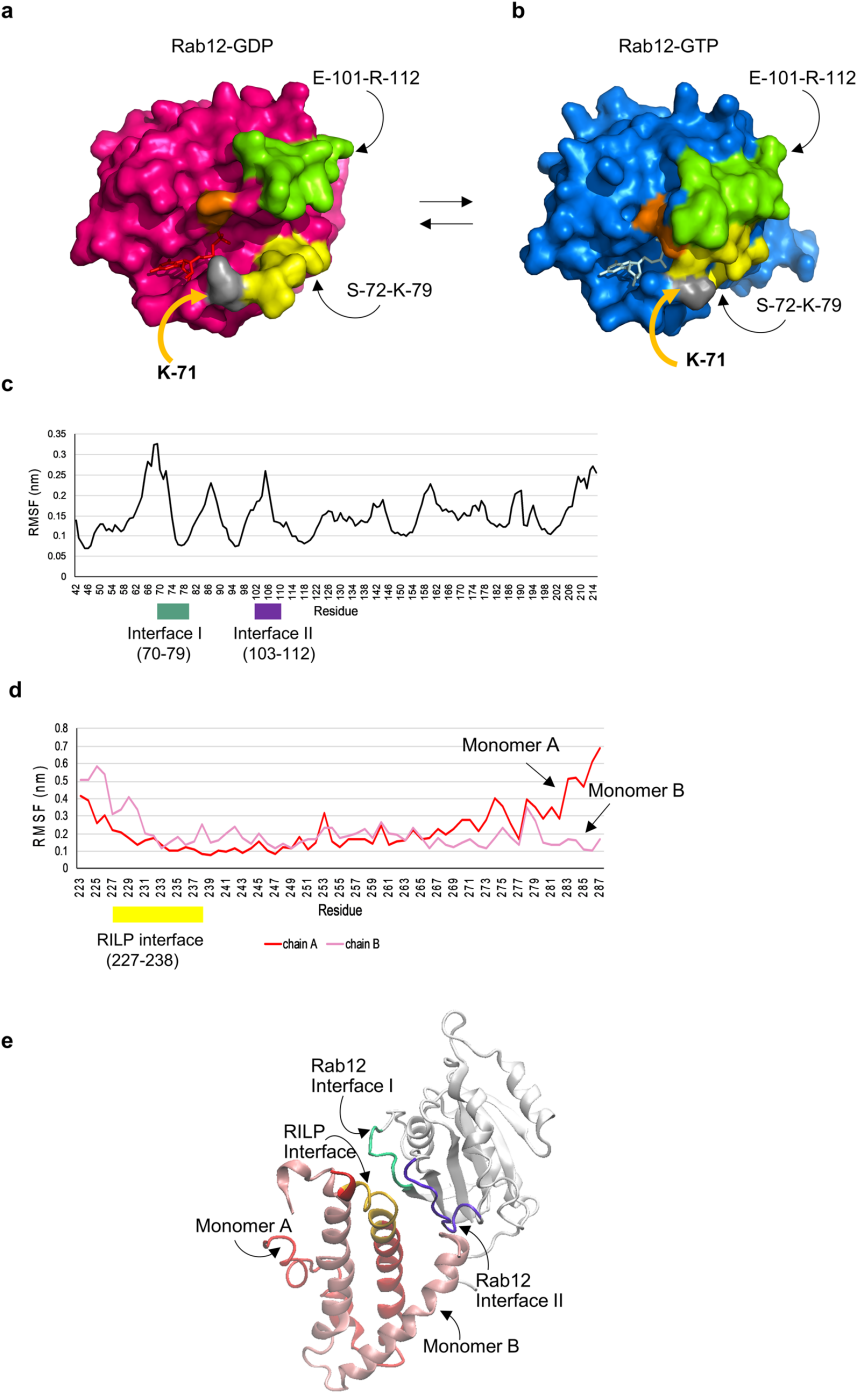
In silico modelling of Rab12 and Rab12-RILP dimer complex structures. (**a**,**b**) In silico model of the structure of GDP-bound (pink) and GTP-bound Rab12 (blue). Highlighted are residues that are affected by the conformational changes that occur during Rab12 activation cycle, K-71 (grey), S-72 to K-79 (yellow) and E-101 to R-112 (green). R-50 is shown in orange. Figures were generated using Pymol Molecular Graphics System, Version 1.2r3pre, Schrödinger, LLC (https://pymol.org/2/). (**c**) RMSF of Rab12 and (**d**) RMSF of the RILP homodimer, during MD simulation. The two predicted Rab12 interfaces are marked in green and purple and the RILP interface in yellow. (**e**) Model for RILP homodimer interaction with GTP-bound Rab12. RILP monomers are shown in red and light pink. Predicted interfaces in Rab12 are shown in green and purple and the predicted interface in RILP in yellow.

In an alternative approach to understand the molecular dynamics of the Rab12-RILP complex and in particular the positioning of K-71 in this context, we generated a computational model of the Rab12-RILP complex. We modelled the GDP-bound conformation of Rab12 using Rab12 X-ray structure (PDB 2IL1) as template^13^, and the GTP-bound conformation on the basis of Rab7 structure (PDB 1YHN)^10^, relying on the fact that the root-mean-square deviation (RMSD) between the atoms of superimposed Rab7 and Rab12 is 0.743 Ǻ, suggesting that their structures are reasonably similar. Based on these models, Rab12 activation is associated with a conformational shift in loops comprising amino acids serine 72 to lysine 79 and glutamic 101 to the arginine at position 112 (Fig. 3a,b), as is reflected in the change in distance between V-74 to F-103, from 14.3 Å in the GDP-bound conformation of Rab12 to 9 Å in its GTP-bound, active conformation, creating a pocket involving the arginine residue at position 50 (Fig. 3a,b). We then docked the active Rab12 model to a RILP homodimer, on the basis of the published structure of the Rab7-RILP dimer complex^10^, and subjected the complex to molecular dynamics (MD) simulations, to predict the modes of Rab12-RILP interactions at atomic resolution. Analyzing the root-mean-square fluctuations (RMSF) of each protein during simulations predicted the existence of two interfaces between Rab12 and the RILP dimer (Fig. 3c,d). The first interface spanned amino acids C-70 to K-79, which include our predicted binding site of Rab12 for its effectors (Fig. 3c,e). The second interface spanned amino acids F-103 to R-112 (Fig. 3c,e), which together with the first interface, are predicted by our model to change location during the Rab12 activation cycle and are therefore expected to interact with RILP in a GTP dependent fashion (Fig. 3a). Restricted mobility was also noted for Rab12 residues L-42 to I-46 (Fig. 3c). However, as these residues reside within the guanine nucleotide binding site, their mobility is likely to be restricted by the binding of GTP, when comparing to the unbound protein.

RILP contains two coiled-coil (CC2) domains, of which the CC2 domain present within its C-terminal half, is conserved within all three members of this family (i.e. the RILP Homology Domain, RHD) (Supplementary figure S2d). This domain was shown to mediate RILP binding to Rab7, Rab34 and Rab36^12^. Consistent with the involvement of the RHD in mediating RILP interactions with Rab GTPases, our model has positioned residues L-227 to K-238, which comprise the RILP RHD, at the Rab12 interface (Fig. 3d,e). In fact, both interfaces of Rab12 were predicted to interact with the RHD of same RILP monomer, while an additional contact was predicted to form between Rab12 and the second RILP monomer (Fig. 3d,e). Therefore, unlike the Rab7-RILP tetrameric complex, that consists of a RILP homodimer complexed to two molecules of Rab7^10^, Rab12 is predicted to form a ternary complex consisting of a RILP homodimer and a single molecule of Rab12.

**Figure 4:**
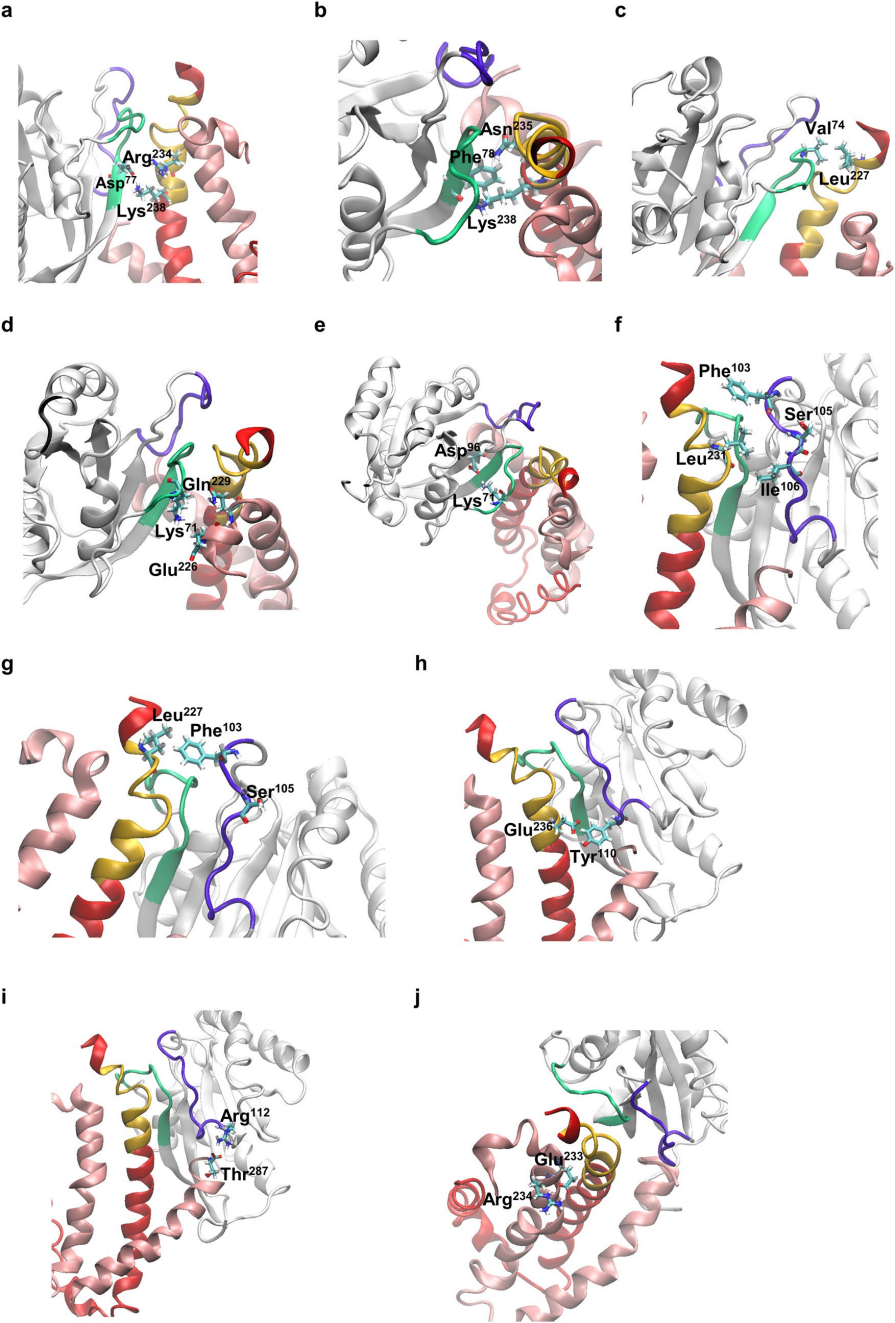
Predicted interactions within the Rab12—RILP complex. (**a**) A medium strength salt bridge is generated between Rab12 Asp-77 and RILP residue Arg-234, and a stronger interaction between Asp-77 and Lys-238 present within RILP RHD (yellow) of same monomer (red). (**b**) A stable interaction occurs between Phe-78 and Lys-238. (**c**) Rab12 Val-74 interacts with Leu-227 of same RILP monomer. (**d**) Rab12 Lys-71 is pulled away from RILP residues Glu-226 and Gln-229. (**e**) Lys-71 forms an intramolecular hydrogen bond with Asp-96. (**f**) Shown are the positional interactions between Phe-103 and Ile-106 of the second Rab12 interface (purple) with RILP residue Leu-231 that resides in RILP RHD (yellow). The relative position of Ser-105 is also depicted. (**g**) Phe-103 also interacts with Leu-227 of same RILP monomer (red). (**h**) A hydrogen bond is formed between Rab12 Tyr-110 and residue Glu-236 at the RHD of same RILP monomer. (**i**) Arg-112 interacts with residue Thr-287 of the second RILP monomer (light pink). (**j**) Glu-233 located in one RILP monomer interacts with residue Arg-234 of the second monomer.

Further analysis of the MD trajectories predicted stable interactions between D-77 that resides in the first interface of the Rab12-RILP complex, and residues R-234 and K-238 of a single RILP monomer (Supplementary Table 1 and Fig. 4a), phenocopying the interaction of Rab7 D-44, the equivalent of Rab12 D-77 in Rab7 (Fig. 2a), with residues R-255 and K-259, the equivalents of mouse R-234 and K-238 in human RILP^10^. MD trajectories also predicted a highly stable interaction between F-78 and RILP residue K-238 and a more labile interaction between this residue and RILP N-235 (Supplementary Table 1 and Fig. 4b), in analogy to the interactions of F-45, the Rab7 equivalent of Rab12 F-78 (Fig. 2a), with N-256 and K-259 residues in human RILP residues^10^. Interestingly, though I-41 of Rab7 is replaced in Rab12 by V-74 (Fig. 2a), and F-248, which in human RILP interacts with I-41^10^, is replaced in mouse RILP by L-227, our MD simulations predicted an analogous stable interaction between Rab12 V-74 and RILP-L227 (Supplementary Table 1 and Fig. 4c). Contradictory to the Rab7-RILP complex, in which Rab7 K-38 plays an important role via its interactions with E-247 and Q-250 in human RILP^10^, no interactions were predicted between Rab12 K-71, the equivalent of Rab7 K-38, and E-226 and Q-229, the equivalent residues in mouse RILP (Supplementary Table 1 and Fig. 4d). In fact, K-71 seemed to be engaged in an intramolecular interaction mediated by a hydrogen bond with D-96 (Supplementary Table 1 and Fig. 4e), thus providing an explanation for the lack of impact of the K-71 mutation on the binding of RILP.

In sharp contrast to the interactions within the first interface of the Rab12-RILP complex, which largely recapitulated the interactions of the first interface of the Rab7-RILP complex, the second interface of the Rab12-RILP complex is unique, sharing no homology with the Rab7-RILP complex. MD trajectories predicted interactions between both F-103 and I-106 of Rab12 and same RILP residue L-231. Hence, during 52% of time of simulation, L-231 was located in close proximity to F-103, while during 41% of time, L-231 was proximal to I-106 (Supplementary Table 1, Fig. 4f). A short-lived interaction, accounting for only 7% of time of simulation, was recorded between L-231 and S-105 of Rab12 (Supplementary Table 1, Fig. 4f). Intriguingly, this amino acid is the site of Rab12 phosphorylation by the Parkinson’s disease-related kinase Leucine-Rich Repeat kinase 2 (LRRK2)^14^, which stimulates Rab12 binding of RILP-L2, but not of RILP-L1^9^. Whether or not LRRK2-mediated phosphorylation of Rab12 affects binding of RILP is presently unknown.

**Figure 5:**
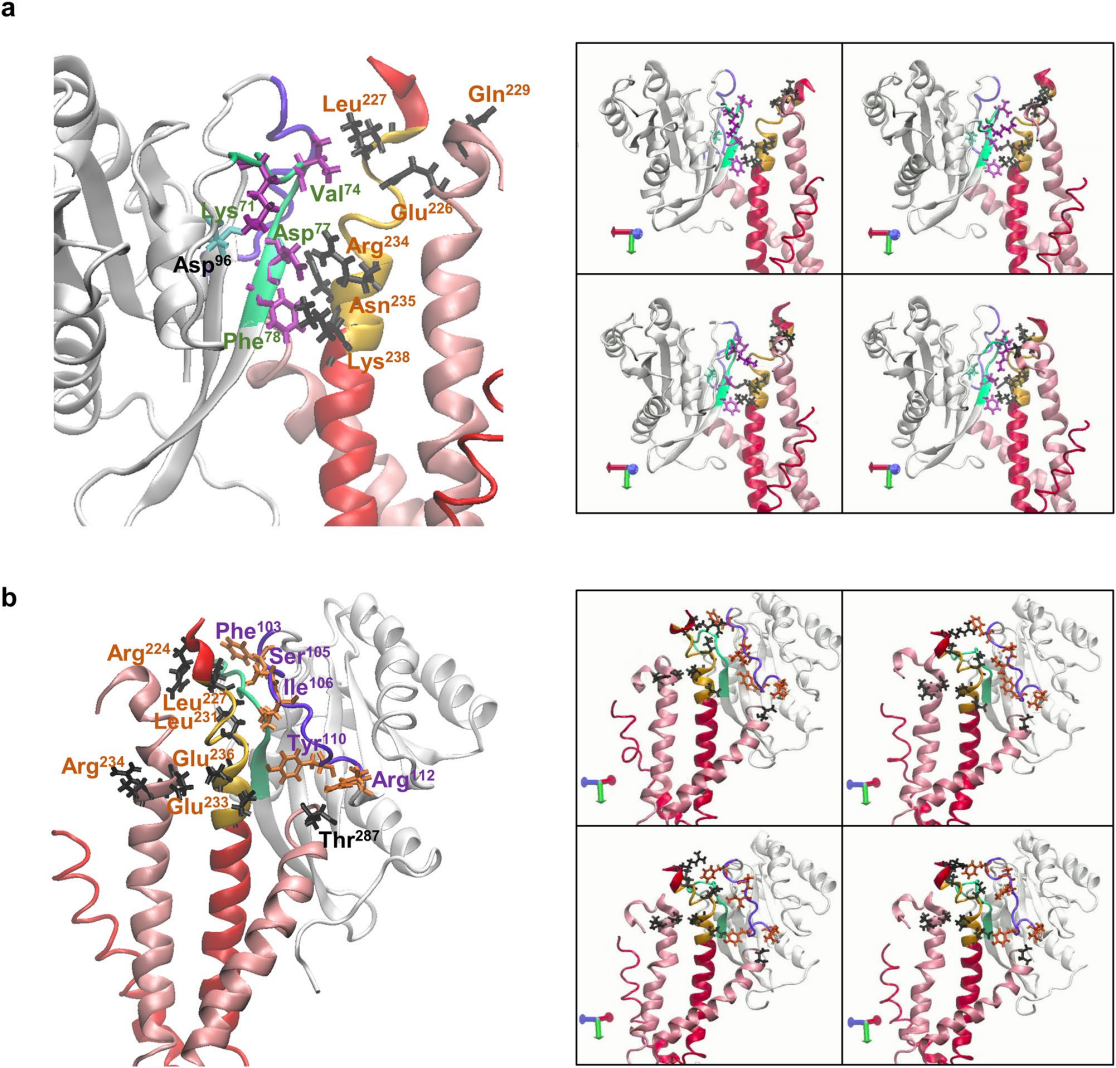
Dynamics of Rab12-RILP interactions. (**a**) Snapshots illustrating the dynamics of interactions within the first interface of the Rab12–RILP complex. Rab12 amino acids that form the first interface (green) are coloured in purple, and RILP RHD (yellow) amino acids that bind Rab12 are coloured in dark grey. RILP monomers are coloured in red and light pink. (**b**) Snapshots illustrating the dynamics of interactions within the second interface of the Rab12–RILP complex. Rab12 amino acids that form the second interface (purple) are coloured in orange, and RILP RHD (yellow) amino acids that bind Rab12 are coloured in dark grey. RILP monomers are coloured in red and light pink.

MD trajectories also disclosed interactions between F-103 and L-227. Thus, when F-103 was not in contact with L-231, it was engaged in an interaction with L-227, which also forms contact with V-74 of the first Rab12 interface (Supplementary Table 1, Fig. 4g). Two additional interactions of the second interface of Rab12 were predicted for Y-110 and RILP residue E-236 (Supplementary Table 1, Fig. 4h), and R-112 with RILP residue T-287 (Supplementary Table 1, Fig. 4i). However, unlike residues F-103, I-106 and Y-110, which form contacts with same RILP monomer (Monomer A, Fig. 4i), R-112 forms a stable hydrogen bond with the threonine residue of the second RILP monomer (Monomer B, Fig. 4i), consistent with the RMSF variability of the C-terminal regions of the two RILP monomers (Fig. 3d). Finally, a strong and stable interaction was predicted between E-233 of monomer A and R-234 of monomer B (Supplementary Table 1, Fig. 4j), implicating these residues in RILP dimerization. Taken together, the simulated model suggests a ternary Rab12-RILP homodimer complex, governed by the RHD of one RILP monomer that associates with two interfaces of Rab12, of which the second interface also associates with the second monomer of the RILP dimer (Fig. 5 and Supplementary Videos 1 and 2).

### Peptides comprising the predicted Rab12 interfaces within the Rab12-RILP complex inhibit RILP binding of Rab12

**Figure 6:**
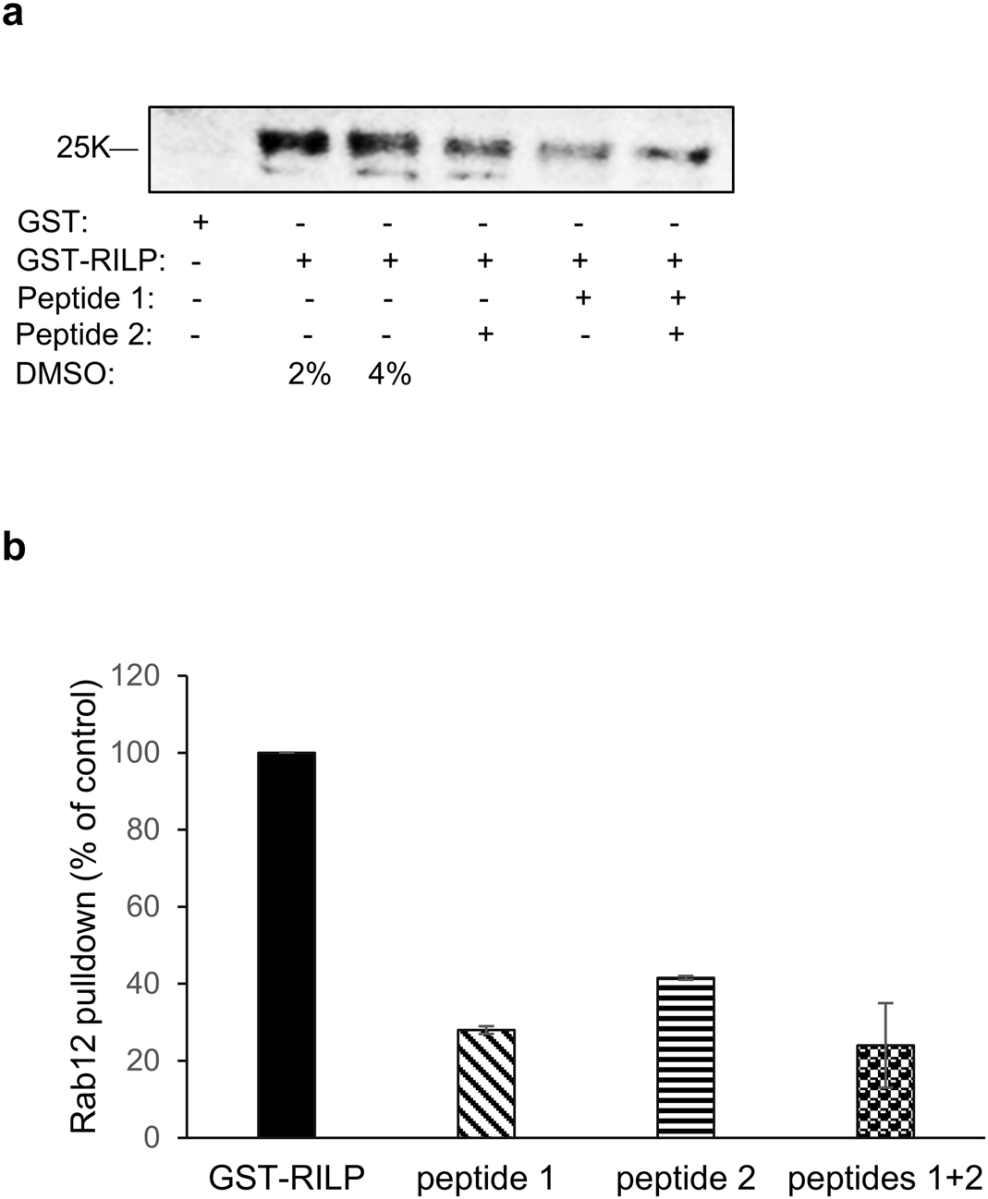
Peptide inhibition of Rab12 interaction with RILP. (**a**) Control GST and GST-RILP (5 μg), immobilized on glutathione agarose beads, were incubated for 4 h at 4 °C with either vehicle (containing 2 or 4% DMSO, as indicated) or 200 μM of peptide 1, peptide 2 or their combination, as indicated, followed by 18 h incubation with RBL cell lysates (500 μg), in the presence of 0.5 mM GTPγS. Bound proteins were resolved by SDS-PAGE and analyzed by immunoblotting with anti Rab12 antibodies. A representative blot is shown. (**b**) The amount of pulled down Rab12 was quantified using the ImageJ software. The results are the average pulldown ± SEM derived from two independent experiments.

To attain independent support to our predicted assignment of Rab12 interfaces I and II as mediators of Rab12 interaction with RILP, we analyzed the impact of peptides comprising sequences within these regions, on RILP ability to pulldown Rab12 from RBL cell lysates. We envisioned that such peptides may compete with endogenous Rab12 for its binding by RILP. Two peptides were synthesized for this purpose, the first comprising amino acids 68 to 82, which reside within the first interface of Rab12 interaction with RILP (i.e. amino acids EACKSTVGVDFKIKT) and the second comprising amino acids 101 to 115, which reside within the second interface (i.e. amino acids ERFNSITSAYYRSAK). The amount of Rab12 pulled down by immobilized GST-RILP was then determined in the absence or presence of the competing peptides. Consistent with our expectation, each of the tested peptides inhibited Rab12 pulldown by 60 to 70%, though neither peptide, nor their combination could eliminate Rab12 binding (Fig. 6). This result is in line with the importance of structural elements in dictating the affinity of Rab12 interaction with RILP, as is evidenced by the dependence of this interaction on GTP.

### Functional analysis of RILP mutants supports the involvement of RILP RHD in Rab12 binding

**Figure 7:**
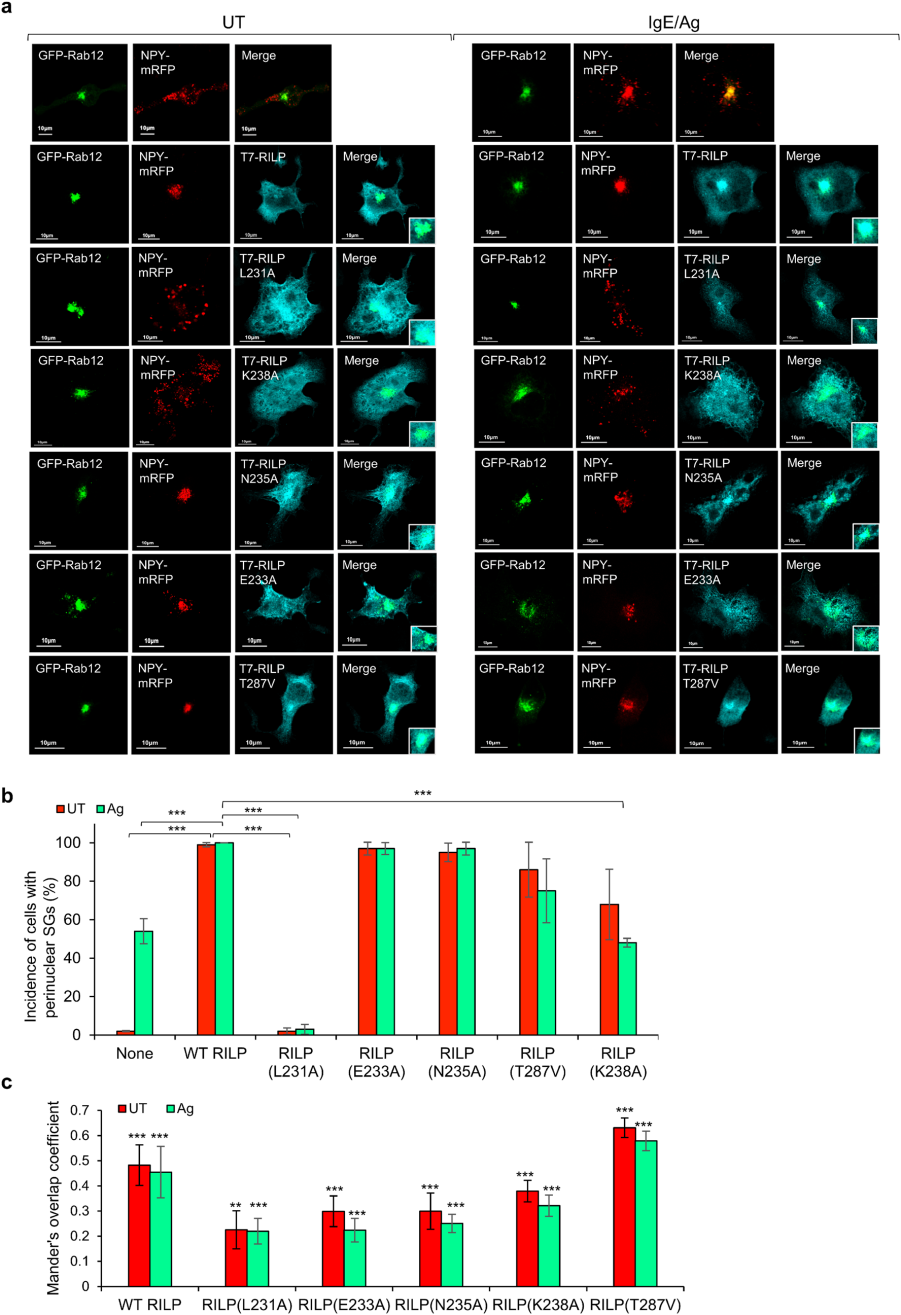
RILP RHD mutants differently affect the SG distribution in MCs, but do not affect Rab12 targeting. (**a**) RBL cells were transiently co-transfected with 15 μg of plasmid encoding NPY-mRFP, 15 μg of pEGFP-C1-Rab12 and 20 μg of either empty vector or pEF-T7-RILP, pEF-T7-RILP(L231A), pEF-T7-RILP(E233A), pEF-T7-RILP(N235A), pEF-T7-RILP(K238A), or pEF-T7-RILP(T287V), as indicated, and grown for 24 h in the presence of IgE (1:512 dilution). Cells were then left untreated (UT) or activated by 50 ng/ml of DNP-HSA (Ag). Cells were subsequently fixed and immunostained with monoclonal antibodies directed against T7, followed by Hilyte Plus 647-conjugated goat anti-mouse IgG. Cells were visualized by confocal microscopy. Bar = 10 μm. (**b**) Quantitative analyses of the incidence of cells that display perinuclear SGs were based on the imaging of 20–35 cells, derived from three separate experiments. A single factor ANOVA was performed followed by a Bonferroni corrected post-hoc T-test. *P* values for untreated cells (UT): ***P[T7-RILP/control] = 3E−9, ***P[T7RILP(L231A)/T7-RILP] = 2E−6; *P* values for IgE/Ag triggered cells: ***P[T7-RILP/control] = 4E−4, ***P[T7RILP(L231A)/T7-RILP] = 3E−6, ***P[T7RILP(K238A)/T7-RILP] = 3E−6. (**c**) Manders’ overlap coefficients for immunostained T7-tagged RILP or RILP mutants and EGFP-Rab12 were determined by calculating the fraction of cyan pixels (RILP signal) that overlap with green pixels (Rab12 signal). Statistical significance was determined with unpaired t test, **P < 0.01; ***P < 0.001.

Though the RHD has previously been shown to mediate RILP interactions with others Rab GTPases, the relative contribution of different residues within this domain to complex formation was distinct and dependent on the Rab type^12^. Therefore, we investigated the impact of RHD mutations shown to impact binding to other Rabs, on the binding of Rab12. Alanine substitution of the leucine residue at position 231 of the RHD, whose mutation impairs RILP interactions with Rab7, Rab34 and Rab36^12^ and which based on our model is predicted to form contact with the second interface of Rab12, significantly (i.e. by 85%) impaired RILP pulldown by immobilized Rab12 (Supplementary figure S3). Alanine substitution of N-235, which impairs binding to Rab34, but does not affect binding to Rab7 or Rab36^12^, resulted in reduced affinity to Rab12, though consistent with our model, which predicted no stable interaction between this RILP residue and Rab12 (Supplementary Table 1), this reduction was not significant (Supplementary figure S3). Mutation of E-233, which based on our model does not directly interact with Rab12, but plays a role in RILP dimerization, significantly (i.e. by 90%) inhibited RILP pulldown by Rab12 (Supplementary figure S3), in a similar manner to its interference with RILP binding to Rab7 and Rab34, but not binding to Rab36^12^. We also tested two additional mutations, comprising alanine substitution of lysine 238, which is predicted by our model to interact with the first interface of Rab12, and valine substitution of threonine 287, predicted to interact with Rab12 interface II in one of RILP monomers (Supplementary Table 1). Both mutants were effectively pulled down by GST-Rab12 (Supplementary figure S3). However, because the in vitro conditions of pulldown assays, which rely on immobilized fusion proteins, may not be sensitive enough to detect weakening of protein–protein interactions that occur in intact cells, and because of the increased degree of non-specific binding to GST that the RILP(K238A) mutant seemed to display (Supplementary figure S3), we also conducted functional assays, seeking for evidence for RILP RHD involvement in the binding of Rab12. To this purpose we relied on the ability of the overexpressed Rab12-RILP complex to recapitulate the phenotype of constitutively active Rab12, which promotes perinuclear clustering of the SGs^2^. Hence, we analyzed how RILP RHD mutations affect the SG distribution in resting MCs, or in cells triggered by immunoglobulin E (IgE) and the respective antigen (IgE/Ag), conditions that stimulate MC degranulation and activation of Rab12^2^. In agreement with our previous results, unlike their distribution throughout the cell in control cells, the SGs, that were labelled with NPY-mRFP, which we have previously shown to serve as a genuine SG reporter^1,2,15^, localized to the perinuclear region in 99% of cells that co-expressed EGFP-Rab12 and T7-tagged RILP (Fig. 7a, b). Also consistent with our previous results, Rab12 localized to the perinuclear region, previously identified as the ERC^2,3^ (Fig. 7a). In consistence with our previous results^2^, in response to the IgE/Ag trigger, the incidence of cells displaying perinuclear SGs was increased (to ~ 50% of cells) and overexpression of wild type RILP further increased their incidence to 100% (Fig. 7a, b). In both resting and IgE/Ag-triggered cells, the increase in perinuclear clustering of the SGs was associated with colocalization of RILP with Rab12, whereby 48% of overexpressed RILP colocalized with Rab12 (Fig. 7a,c). In sharp contrast, regardless to whether triggered or not, the SGs remained scattered in 97% of cells that overexpressed Rab12 and the RILP(L231A) mutant (Fig. 7a,b), which also showed significantly reduced (22%) colocalization with Rab12 (Fig. 7a,c). These results therefore firmly supported the critical role played by this residue in Rab12-RILP complex formation, in line with its central role in mediating RILP interactions also with its other Rab partners^12^. In contrast, RILP (T287V), which bound Rab12 in the pulldown assays, and RILP(N235A), which displayed reduced binding (Supplementary figure S3), maintained functionality, both leading to SG clustering in either resting or triggered cells (Fig. 7a,b), though colocalization of the latter mutant with Rab12 was significantly reduced (Fig. 7a,c). Surprisingly, RILP(E233A), which like RILP(L231A), has lost its ability to bind Rab12 in pulldown assays (Supplementary figure S3), successfully enforced perinuclear clustering of the SGs in all cells (Fig. 7a,b). These results therefore suggest that a protein, other than Rab12, may intervene between the dynein-RILP complex and the SGs to drive their minus end transport. Such a protein may either function redundantly with Rab12, or compensate for the absence of Rab12-RILP interaction. In agreement with this notion, RILP(E233A) showed significant reduction in colocalization with Rab12 in either resting or triggered cells (Fig. 7a,c). Despite its binding by Rab12 in pulldown assays (Supplementary figure S3), the RILP(K238A) mutant stimulated SG clustering in only 68% of resting cells and only 48% of IgE/Ag-tiggered cells, in which the Rab12-RILP complex needs to counteract the plus end transport of the SGs, that is enhanced by the IgE/Ag trigger^16^. In the remaining cells, the SGs were scattered despite the clear expression of this mutant (Fig. 7a,b). Furthermore, this mutant displayed a significant reduction in its colocalization with Rab12 (Fig. 7a,c), implying that its interaction with Rab12 was significantly reduced in intact cells. Therefore, taken together, these results strongly support the role of RILP RHD in mediating RILP interactions with interfaces I and II of Rab12.

### Perinuclear targeting of Rab12 does not depend on Rab12 interactions with its RILP family effectors

**Figure 8:**
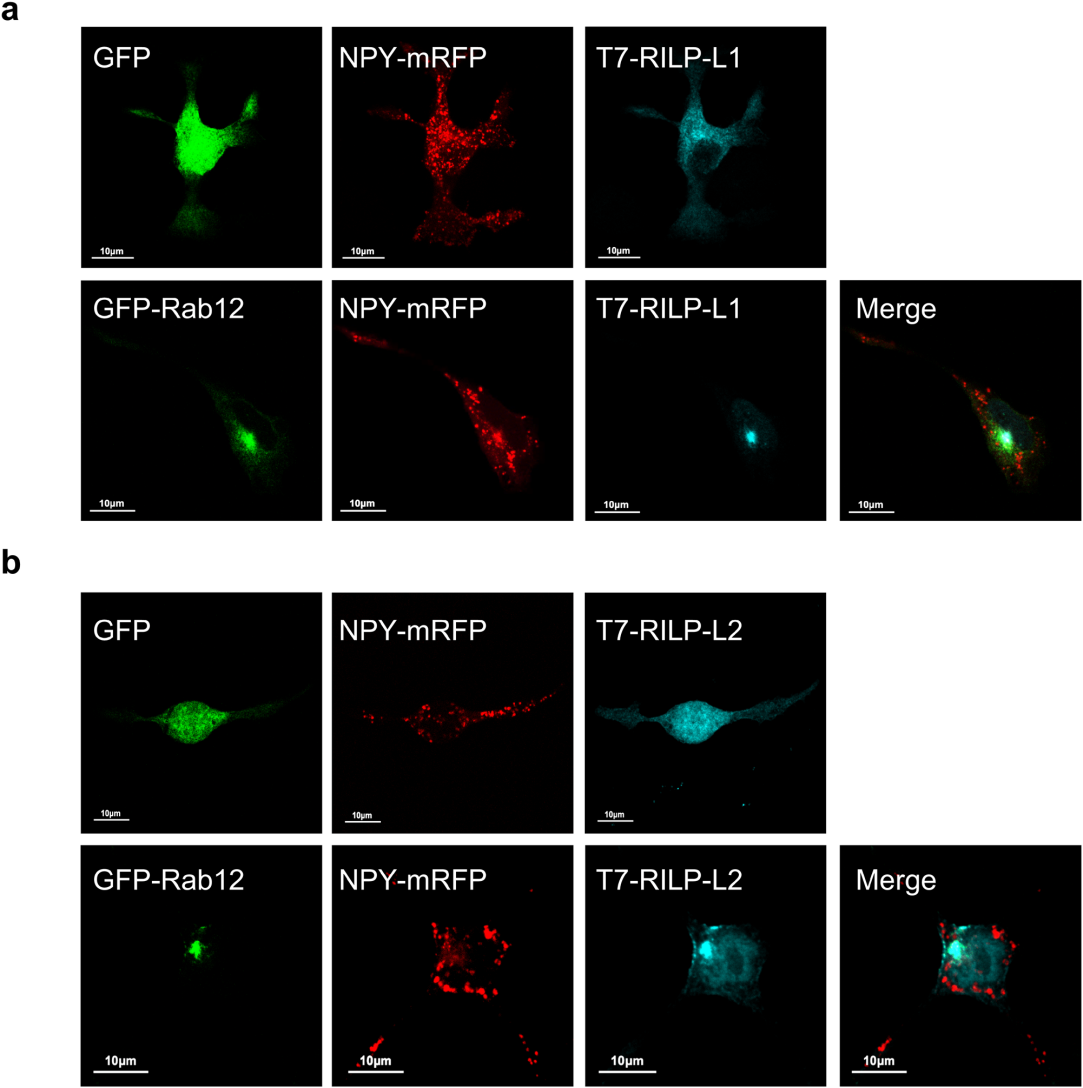
Rab12 recruits RILP-L1 and RILP-L2 to their perinuclear location. RBL cells were transiently co-transfected with 15 μg of plasmid encoding NPY-mRFP, 20 μg of either pEF-T7-RILP-L1 (**a**) or pEF-T7-RILPL-2 (**b**) and 15 μg of either pEGFP-C1 or pEGFP-C1-Rab12, as indicated. After 24 h, cells were fixed and immunostained with monoclonal antibodies directed against T7, followed by Hilyte Plus 647-conjugated goat anti-mouse IgG. Cells were visualized by confocal microscopy. Bar = 10 μm.

We noticed that Rab12 maintained its perinuclear location irrespectively to whether co-expressed with wild type RILP, or with non-interacting RILP RHD mutants (Fig. 7a). These results suggested that RILP plays no role in Rab12 targeting to the ERC. Since RILP-L1 was shown to mediate targeting of Rab10 to its pericentriolar location^17^, we asked whether RILP-L1 or RILP-L2, the other effectors of Rab12, might be responsible for its cellular targeting. However, while both RILP-L1 and RILP-L2 localized to the cytosol when overexpressed in the RBL cells, with EGFP, as a control, these effectors redistributed from the cytosol to the perinuclear region, when co-expressed with EGFP-Rab12 (Fig. 8a,b). Therefore, while these results confirmed Rab12 interactions with these effectors in intact cells, they also implicated Rab12 in the targeting of RILP-L1 and RILP-L2 to pericentriolar membranes, rather than the other way around. Therefore, Rab12 acquires its ERC location independently of its interactions with RILP family members, but it may play a role in their cellular targeting. Notably, co-expression of Rab12 with neither RILP-L1 nor with RILP-L2 had any impact on the cellular distribution of the SGs (Fig. 8a,b), consistent with their lack of a dynein binding domain^18^.

## Discussion

All three members of the RILP family of proteins have been shown to interact with the small GTPase Rab12, though the precise modes of their interactions, role and regulation are still by largely poorly resolved. Towards the understanding of the underlying mechanisms of Rab12 interactions, here we mainly focused on Rab12 interactions with RILP, which we have recently shown to mediate Rab12-driven, and microtubule dependent, minus-end transport of the SGs in MCs^2^. Three important findings emerged from the present study. First, we show that RILP interacts with Rab12 independently of RILP-L1 or RILP-L2. We show that although all members of the RILP family can homodimerize, neither of these proteins forms heterodimers with other family members. This finding therefore excludes the possibility of RILP interaction with Rab12 via a complex with RILP-L1, which is the only RILP family member that was recognized as such during a yeast two hybrid screen^12^. The reason for this discrepancy may reflect distinct dependencies of Rab12-effector interactions on posttranslational modifications. Rab12 was recently identified as a physiological substrate of LRRK2 and its phosphorylation by this kinase stimulated its interaction with RILP-L2, but not its interaction with RILP-L1^9^. Thus, we envision that posttranslational modifications of Rab12 may also regulate its interaction with RILP. This notion gains support from our simulated model of the Rab12-RILP complex, which positions Rab12 residue S105, the phosphorylation site of Rab12 by LRRK2^14^, at one of Rab12-RILP interfaces. Our future studies will investigate this possibility.

We also delineated the binding site of Rab12 for RILP-L1 and RILP-L2, and identified the lysine residue at position 71, as critical for these interactions. To our surprise, though we chose this lysine as candidate on the basis of its analogous position to the lysine residues implicated in RILP binding by Rab7 and Rab34, replacing this lysine by arginine, which has completely abrogated the pulldown of either RILP-L1 or RILP-L2 by Rab12, has failed to affect Rab12 capacity to pull down RILP. This observation prompted us to understand the structural events that occur during Rab12 interaction with RILP, for which we employed in silico modelling that allowed us to gain structural insights at atomic resolution. Intriguingly, although our model was built on the basis of the resolved x-ray structure of the Rab7- RILP dimer complex^10^, molecular dynamics simulations, based on an energy minimized complex structure, revealed some similarities between the Rab12 and Rab7-RILP complexes, but have also outlined significant differences. Unlike the symmetric Rab7-RILP complex, which consists of a central RILP homodimer and two molecules of GTP-bound Rab7, each binding to a single RILP monomer^10^, our model predicts a ternary protein complex between a homodimer of RILP and a single molecule of GTP-bound Rab12 that interacts with both RILP monomers. This ternary complex is held together via multiple bonds that encompass two interfaces in Rab12, that bind to the RHD of one RILP monomer, and a third contact site between an amino acid within the second interface of Rab12 and the second RILP monomer. The first interface of Rab12 interaction with RILP largely replicates the first interface of RILP interactions with Rab7. In both cases, this interface involves the Rab switch I region, comprising amino acids cysteine 70 to leucine 79 in Rab12, which is implicated in Rab effector binding, when bound to GTP. Though exceptional in this regard is the contribution of the lysine residue within the first interface, i.e. K-38 in Rab7, which contributes significantly to Rab7 interaction with RILP, unlike K-71 of Rab12, which is dispensable for Rab12 interaction with RILP, but is rather involved in an intramolecular interaction, mediated by a hydrogen bond with the aspartate residue at position 96, which pulls lysine 71 away from the RILP complex. Also, in analogy to the Rab7-RILP complex, the first interface of the Rab12-RILP complex, includes RILP RHD, which was also implicated in mediating RILP interactions with Rab34 and Rab36^12^.

In sharp contrast, though both Rab7 and Rab12 interact with RILP via an additional interface, a number of important differences distinguish between the two. First, the second predicted interface of Rab12, that spans residues phenylalanine 103 to arginine 112, resides at the conserved RabF3 and RabF4 regions, which similarly to switch I, and consistent with our model, undergo positional changes during the Rab12 activation cycle^19^. This is different from the second interface of Rab7 that resides in its hypervariable regions RabSF1 and RabSF4^10^. Therefore, unlike Rab7, the second interface of Rab12 is also predicted to bind RILP in a GTP-dependent fashion. Second, unlike Rab7, the second interface of Rab12 also involves the RILP RHD, similarly to the first interface. In this respect, Rab12 interaction with RILP replicates RILP RHD interaction with Rab36, that involves the switch II region of Rab36^12^. The assignment of switch I and II as interfaces within the Rab12-RILP complex has gained independent support from the following findings: First, peptides comprising amino acids predicted to form contact with RILP RHD in either interface, effectively inhibited the pulldown of Rab12 by immobilized GST-RILP. Second, mutations in RILP residues, predicted to interact with either interface I (lysine 238) or interface II (leucine 231), inhibited the functional activity of the Rab12-RILP complex in driving minus end transport of the SGs and leading to their perinuclear accumulation. Such mutants also displayed reduced colocalization with Rab12 in either resting cells, or cells that were activated by an IgE/Ag trigger, conditions that increase Rab12 activation^2^. The second interface of Rab12 was also predicted to form contact with the second monomer of the RILP dimer by forming a hydrogen bond between arginine 112 and the threonine residue at position 287 of RILP. However, replacement of this threonine by valine has failed to impact the pulldown of this mutant by Rab12, neither did it affect the SG distribution. Nevertheless, given the multiple interactions that take place between the two Rab12 interfaces and RILP RHD, the relative contribution of this contact site might be too low to impact the function of the overexpressed complex.

Surprisingly, though a mutation in the glutamic residue at position 233, which is predicted to mediate RILP dimerization, has impaired RILP binding by Rab12 in pulldown and colocalization assays, this mutant was able to cluster the SGs. This mutant binds Rab36, which based on our Rab screen, induces perinuclear clustering of the SGs^1^. Therefore, we are prompted to suggest that Rab12 and Rab36 may either function redundantly or play complementary roles in controlling MC SG transport. In this context, it is interesting to note that unlike overexpressed Rab12, that clusters the SGs only in its constitutively active conformation or in triggered cells, overexpressed Rab36 clusters the SGs also in its wild type form and in resting cells^1,2^. Since MC SGs move bidirectionally also in resting cells^2,20^, it is tempting to speculate that Rab36 drives their retrograde transport in resting cells, while Rab12 drives their transport in activated cells, in its capacity as negative regulator of MC secretion^2^.

Finally, we show that Rab12 acquires its perinuclear location, previously identified as the ERC, regardless to its interactions with its effectors. This is illustrated in the fact that Rab12 is perinuclear also in cells that overexpress the RILP RHD mutants, thus excluding its interaction with RILP in its targeting to the ERC. Similarly, both RILP-L1 and RILP-L2 are primarily cytosolic when overexpressed in the absence of Rab12, but translocate to the perinuclear region, colocalizing with Rab12, in its presence. This contrasts the Rab-effector relationship between RILP-L1 and Rab10, where RILP-L1 localizes to pericentriolar membranes and enhances the accumulation of phosphorylated Rab10 at this site^17^. It will be interesting to explore if Rab12 initiates a Rab cascade by recruiting RILP-L1, that in turn recruits phospho-Rab10. In this case, Rab12 may represent a missing link in the crosstalk between the ERC, centrosome and primary cilia.

In conclusion, by combining in silico modelling with functional mutational analyses, we provide structural insights into the mode of interactions of Rab12 with its RILP effector, on the basis of which, new tools could be developed for further understanding of the regulation and function of the interactions between RILP family members and Rab12.

## Methods

### Antibodies and reagents

Monoclonal Anti-T7 IgG (Cat #69522-3) was from Novagen. Hilyte Plus 647-conjugated goat anti-mouse IgG (Cat #AS-61057-05-H647) was from Anaspec (Fremont, CA). Horseradish-peroxidase (HRP)–conjugated goat anti–rabbit (Cat # 111-035-003) or anti–mouse (Cat # 115-035-166) IgG were from Jackson ImmunoResearch Laboratories (West Grove, PA). Polyclonal Anti-Rab12 (Cat # 18843-1-AP) was from Proteintech (Chicago, IL). Hybridoma IgE was from Myeloma IgE producing cells (clone Hi 26.86), a kind gift from Dr. Ullrich Blank (Inserm, Paris, France). Glutathione-Agarose (Cat # G4510) and guanosine 5′-[γ-thio] thriphosphate (Cat # G8634) were from Sigma-Aldrich (St. Louis, MO). Dinitrophenyl (DNP)-conjugated human serum albumin (Cat #A6661) was from Sigma-Aldrich Chemicals Co (St. Louis, MO). Peptides 1 and 2 were synthesized by the Blavatnik Center for Drug Discovery, at Tel Aviv University.

### Plasmids used in this study

pEF-T7-RILP, pEF-T7-RILP-L1, pEF-T7-RILP-L2, pEF-T7-RILP(L231A), pEF-T7-RILP(E233A) and pEF-T7-RILP(N235A) were prepared as described in^12^. pGAD-C1-RILP, pGAD-C1-RILP-L1, and pGAD-C1-RILP-L2 were prepared as described in^12^. cDNAs of mouse RILP, RILP-L1, and RILP-L2 were also subcloned into the pGBD-C1 vector^21^. pEF-T7-RILP(K238A) and pEF-T7-RILP(T287V) were prepared by the PCR sewing technique^22^ using pEF-T7-RILP as a template. pEGFP-C1-Rab12 was prepared as described in^3^. pGEX-4T-3-Rab12 was prepared as described in^8^. pGEX-4T-3-Rab12(K71R) was prepared by site-directed mutagenesis, using the Q5 site-directed mutagenesis kit (NEB, Cat # E0554S) and the following primers: Forward primer: GAGGCCTGCAgGTCCACCGTG, Reverse primer: GCAGAACGTGTCGTCGTG. cDNAs of mouse RILP, RILP-L1, and RILP-L2^12^ were subcloned into the pGEX-4T-3 vector (GE Healthcare, Chicago, IL; named pGEX-4T-3-RILP, pGEX-4T-3-RILP-L1, and pGEX-4T-3-RILP-L2) and pEGFP-C1 vector (Clontech/Takara Bio, Shiga, Japan; named pEGFP-C1-RILP, pEGFP-C1-RILP-L1, and pEGFP-C1-RILP-L2).

### Cell culture

RBL cells (the RBL-2H3 subline) were maintained as adherent cultures in low glucose DMEM, supplemented with 10% FBS, 2 mM L-glutamine, 100 μg/ml streptomycin and 100 units/ml penicillin in a humidified incubator of 5% CO_2_ at 37 °C.

### Transient transfection of RBL cells

Transient transfection of RBL cells was performed as previously described^2^. Briefly, RBL cells (1.5 × 10^7^) were transfected with a total of 30–60 μg of cDNAs by electroporation at 300 V for 20 ms using an ECM 830 electroporator (BTX, USA). The cells were immediately replated in tissue culture dishes containing growth medium for the desired time periods.

### Cell activation

RBL cells were seeded onto 24-well plates containing 12-mm round glass coverslips (thick #1; Thermo Scientific, Menzel-Gläser, Saarbrücken, Germany), at 4 × 10^5^ cells/wells. Cells were grown overnight with or without 1:512 dilution of supernatant derived from a DNP specific IgE secreting Hybridoma. After three washes in Tyrode’s buffer (10 mM Hepes, pH 7.4, 130 mM NaCl, 5 mM KCl, 1.8 mM CaCl_2_, 1 mM MgCl_2_, 5.6 mM glucose, and 0.1% BSA), cells were either left untreated or triggered for 30 min at 37 °C with 50 ng/ml DNP-HSA (Ag). Cells were washed three times with PBS and processed for confocal analyses.

### Immunostaining and confocal analyses

Immunostaining and confocal analyses were performed as previously described^2^. Briefly, cells were fixed for 20 min at room temperature with 4% paraformaldehyde in PBS. Cells were then permeabilized for 20 min at room temperature with 0.1% Triton X-100, 5% FBS, and 2% BSA diluted in PBS. Cells were subsequently incubated for 1 h at room temperature with the primary Abs, followed by three washes and 1 h incubation with the appropriate secondary Abs. After washing, the cells were mounted (Golden Bridge Life Science, Mukilteo City, WA) and analyzed using a LEICA SP8 STED high resolution laser scanning confocal microscope (Leica, Wetzlar, Germany) using a 63 oil/1.4 numerical aperture objective. Colocalization analysis of immunostained T7-tagged RILP or RILP RHD mutants with EGFP-Rab12 was quantified by calculating the Mander’s overlap coefficients using the ImageJ software.

### Co-immunoprecipitation assays

RBL cell lysates (500 μg) prepared in buffer A (50 mM Hepes pH 7.4, 250 mM NaCl, 1 mM MgCl_2_, 1% Triton X-100, protease inhibitor mixture, 1 mM PMSF, 2 mM Na_3_VO_4_) were incubated overnight at 4 °C with either rabbit polyclonal anti-GFP antibodies (2 μg) or mouse monoclonal anti-T7 antibodies (1 μg). Protein G plus protein A-Sepharose (50% v/v) was then added for 1.5 h, at 4 °C. Immune complexes were collected, washed three times with buffer B (50 mM Hepes pH 7.4, 150 mM NaCl, 1 mM MgCl_2_, 0.2% Triton X-100, protease inhibitor mixture, 1 mM PMSF, 2 mM Na_3_VO_4_), and resuspended in 1X sample buffer, and boiled for 7 min. Proteins were resolved by SDS-PAGE and analyzed by immunoblotting with the desired antibodies.

### Pulldown assays

Pulldown assays were performed as previously described^2^. Briefly, 20 μg of GST fusion proteins or control GST immobilized on Glutathione Agarose beads were incubated for 18 h at 4 °C with RBL cell lysates (500 µg) prepared in buffer C (50 mM Hepes pH 7.4, 150 mM NaCl, 1 mM MgCl_2_, 1% TritonX100, 1 mM PMSF, protease inhibitor mixture, 2 mM Na_3_VO4) in the presence of 0.5 mM GTPγS. At the end of the incubation period, beads were sedimented by centrifugation at 5000 × *g* for 5 min at 4 °C, washed 4 times with buffer B, and finally suspended in sample buffer, boiled for 7 min, and subjected to SDS-PAGE and immunoblotting.

### Yeast two-hybrid assays

Yeast two hybrid assays were performed as described in^23^. In brief, yeast cells containing pGAD-C1-RILP (-RILP-L1, or RILP-L2) and pGBD-C1-RILP (-RILP-L1, or RILP-L2) plasmids were streaked and incubated at 30 °C on a synthetic complete (SC) medium lacking Leu and Trp (SC-LW) for two days and an SC medium lacking adenine, His, Leu, and Trp (SC-AHLW; selection medium) for one week.

### Western blot analysis

Western blot analyses were performed as previously described^2^. Briefly, samples were separated by SDS-PAGE using 10–12% polyacrylamide gels and electrophoretically transferred to nitrocellulose membranes. Blots were blocked for 20 min in TBST (10 mM Tris-HCl, pH 8.0, 150 mM NaCl, and 0.05% Tween 20) containing 5% skim-milk, followed by overnight incubation at 4 °C with the desired primary Abs. Blots were washed three times and incubated for 1 h at room temperature with HRP-conjugated secondary Ab. Immunoreactive bands were visualized by the ECL method according to standard procedures. The intensity of the immunoreactive bands was quantified using the ImageJ software (https://imagej.nih.gov/ij/index.html).

### Molecular dynamics

The GDP bound conformation of Rab12 was modeled using swiss model^24^ with Rab12 X-RAY structure (PDB 2IL1) as a template. Missing loop coordinates (residues 64–77) was completed using Ypt1, Rab GTPase from yeast (PDB 2BC6)^25^ as a template. The GTP bound conformation was modeled using HHPRED^26^ and Modeller^27^ with Rab7 X-RAY structure (PDB 1YHN)^10^ as a template. Reconstructing RILP dimer was done using the crystal symmetry of RILP structure bound to Rab7 (PDB 1YHN) with Pymol. Docking RILP dimer to Rab12 models was done using GRAMM-X^28^ and Patchdock^29,30^ followed by the refinement docking tools Firedock^31^ and ZDOCK^32^. MD simulation was conducted for 162 ns using the GROMACS package, version 2018.2 (http://www.gromacs.org/).

### Statistical analysis

Data are expressed as means ± SEM. The *P* values were determined by an unpaired two-tailed Student’s *t* test or by ANOVA followed by Bonferroni corrected post-hoc t-test, for multiple comparisons.

## Supplementary Information

Supplementary Information.

Supplementary Video 1.

Supplementary Video 2.
