# Supplementary material for: Biochemical and structural insights into Rab12 interactions with RILP and its family members
Source: Sci Rep. 2021 May 13;11:10317. doi: 10.1038/s41598-021-89394-y (PMC8119438; doi:10.1038/s41598-021-89394-y)
Supplement: Supplementary file 1 — Supplementary Information. [file 41598_2021_89394_MOESM1_ESM.pdf]

Supplementary Information for

Biochemical and structural insights into Rab12 interactions with RILP and its family members

Jana Omar[1], Efrat Rosenbaum[1], Adi Efergan[1], Bayan Abu Sneineh[1], Advа Yeheskel[2], Yuto Maruta[3], Mitsunori Fukuda[3] and Ronit Sagi-Eisenberg[1*].

[1]Department of Cell and Developmental Biology, Sackler Faculty of Medicine, Tel Aviv University, Tel Aviv 69978, Israel, [2]Bioinformatics Unit, Faculty of Life Sciences Tel-Aviv University, Tel Aviv 69978, Israel, [3]Laboratory of Membrane Trafficking Mechanisms, Department of Integrative Life Sciences, Graduate School of Life Sciences, Tohoku University, Aobayama, Aoba-ku, Sendai, Miyagi 980-8578, Japan

*Corresponding author: e-mail address histol3@tauex.tau.ac.il

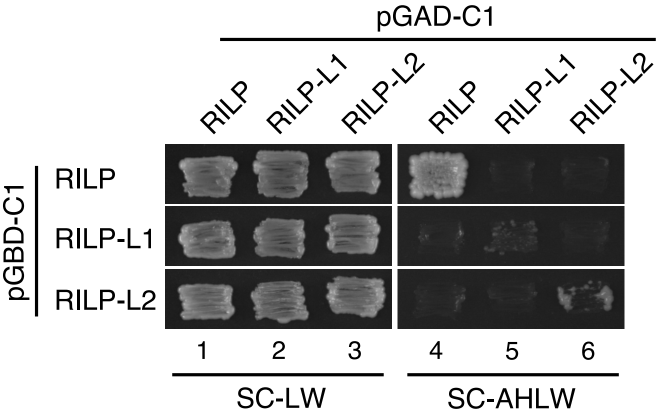

Supplementary Figure S1. Homo- and hetero-dimerization activity of the RILP family members as revealed by yeast two-hybrid assays. Yeast cells containing pGBD plasmid expressing RILP (RILP-L1, or RILP-L2) and pGAD plasmid expressing RILP (RILP-L1, or RILP-L2). The yeast cells were streaked and incubated at 30°C on SC-LW (left panels) and SC-AHLW (right panels).

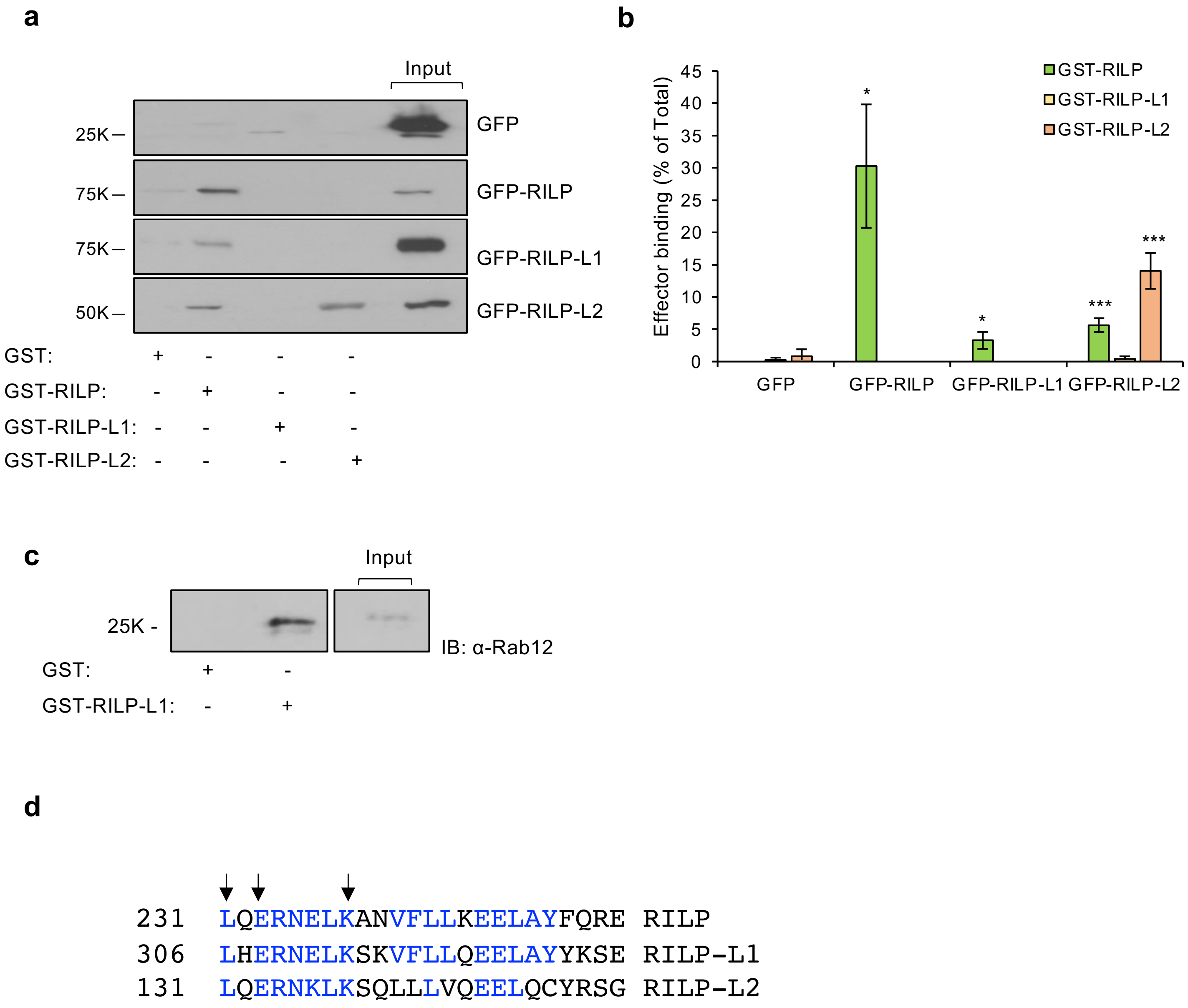

Supplementary Figure S2. Homo- and hetero-dimerization activity of the RILP family members as revealed by pulldown assays.

(**a**) GST, GST-RILP, GST RILP-L1 or GST-RILP-L2 (20 µg) were immobilized on glutathione agarose beads and incubated for 18 h at 4°C with RBL cell lysates (500 µg) derived of cells transfected with 35 µg of either pEGFP-RILP, pEGFP-RILP-L1 or pEGFP-RILP-L2, as indicated. Bound proteins were eluted by sample buffer, resolved on SDS-PAGE and analysed by western blot, using monoclonal anti-GFP antibodies. Input =10% of total protein. Representative blots are shown. (**b**) The amount of pulled down proteins was quantified using the ImageJ software. Results are the average pulldown ± SEM derived from five independent experiments.**P*(GFP-RILP/GST-RILP) = 0.0149, **P*(GFP RILP-L1/GST-RILP) = 0.0327, ***P*(GFP-RILP-L2/GST-RILP) = 0.0024, ***P*(GFP-RILP-L2/GST-RILP-L2) = 0.0021. (**c**) RBL cell lysate (500 µg) was incubated for 18 h at 4°C in the presence of 0.5 mM GTPγS with 20 µg of immobilized GST or GST-RILP-L1. Bound proteins were eluted by sample buffer and analysed by SDS-PAGE and immunoblotting with antibodies directed against Rab12. Input =10% of total protein. A representative blot is shown. (**c**) Sequence alignment of the RHD of mouse RILP, RILP-L1 and RILP-L2. The arrows point to the amino acids that are involved in the binding of Rab12 by RILP.

a

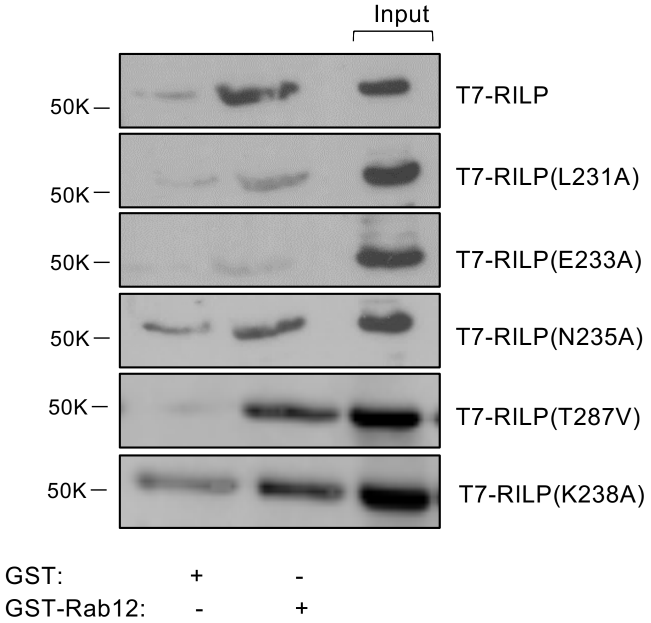

b

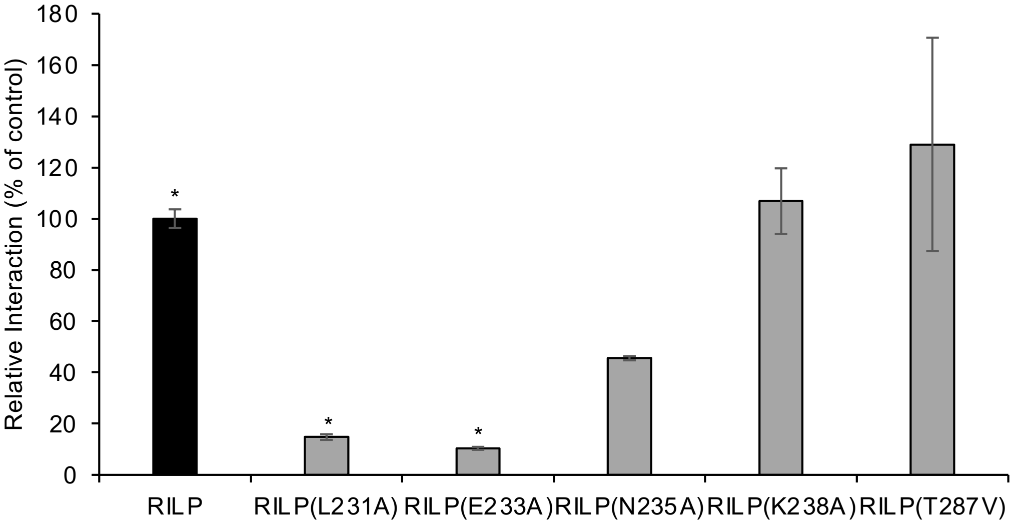

Supplementary Figure S3. Mutational analysis supports RILP RHD involvement in mediating Rab12 binding.

(**a**) RBL cell lysates (500 µg) derived from RBL cells transfected with 35 µg of plasmids encoding T7-tagged RILP, or the indicated T7-tagged RILP RHD mutants, were incubated for 18 h at 4°C, in the presence of 0.5 mM GTPγS with 20 µg of immobilized GST or GST-Rab12. Bound proteins were eluted by sample buffer, and analyzed by SDS-PAGE and immunoblotting, using monoclonal antibodies directed against T7. Input =10% of total protein. A representative blot is shown. (**b**) The amount of pulled down proteins was quantified using the ImageJ software. The results, presented as percentage of pulldown of wild type RILP, are the average pulldown ± SEM derived from three independent experiments. **P*[(T7-RILP/T7-RILP(L231A)] = 0.0480, **P*[T7-RILP/T7-RILP(E233A)] = 0.0498.

Rab12	RILP(A)	RILP(B)	Distance (nm)	Mode of interaction	Time of simulation (%)
K-71 (NZ)	E-226 (OE1/OE2)	-		unstable	
K-71 (NZ)	Q-229 (NH1/NH2)	-		unstable	
K-71 (NZ) D-96 (OD1/OD2)	-	-	<0.3	Hydrogen bond	59.7
V-74 (CG)	L-227 (CD)	-	0.3	Van Der Waals	60
D-77 (OD1/OD2)	K-238 (NZ)	-	<0.35	Strong salt bridge	99
D-77 (OD1/OD2)	R-234 (NH1/NH2)	-	<0.5	Medium strength salt bridge	96
F-78	N-235 (ND2)	-		No interaction	
F-78 (CG)	K-238 (NZ)	-		Stable cation-pi interaction	99.8
F-103 (CG)	L-227 (CD1/CD1)	-	0.33	Van Der Waals	60
F-103	R-224	-		No interaction	
F-103 (phenyl ring)	L-231 (CD1/CD2)	-	0.3	Van Der Waals	52.4
S-105	L-227	-		No interaction	
S-105 (OG)	L-231 (O/N)	-		No interaction	
I-106 (CD)	L-231 (CD1/CD2)	-	0.32	Van Der Waals	41
Y-110 (OH)	E-236 (OE1/OE2)	-	<0.3	Hydrogen bond	56.5
R-112 (NH1/NH2)	-	T-287 (OD1/OD2)	<0.3	Hydrogen bond	89
-	E-233 (OE1/OE1)	R-234 (NH1/NH2)	<0.3	Strong salt bridge	97.7

Supplementary Table 1: Rab12 and RILP contacts along the MD trajectories.
The table presents the type of bonds that are generated between atoms within Rab12 RILP monomer atoms. The percentage of time that the contacts are maintained along the trajectory are indicated.

Supplementary Videos

Video 1. Dynamics of interactions within the first interface of the Rab12 – RILP complex

Molecular dynamics simulations of the Rab12-RILP complex showing the interactions between Rab12 amino acids that form the first interface (green) and coloured in purple, and their interacting RILP RHD (yellow) amino acids, coloured in dark grey. RILP monomers are coloured in red and light pink.

Video 2. Dynamics of interactions within the second interface of the Rab12 – RILP complex

Molecular dynamics simulations of the Rab12-RILP homodimer complex showing the interactions between Rab12 amino acids that form the second interface (purple) and coloured in orange, and their interacting RILP RHD (yellow) amino acids, coloured in dark grey. RILP monomers are coloured in red and light pink.

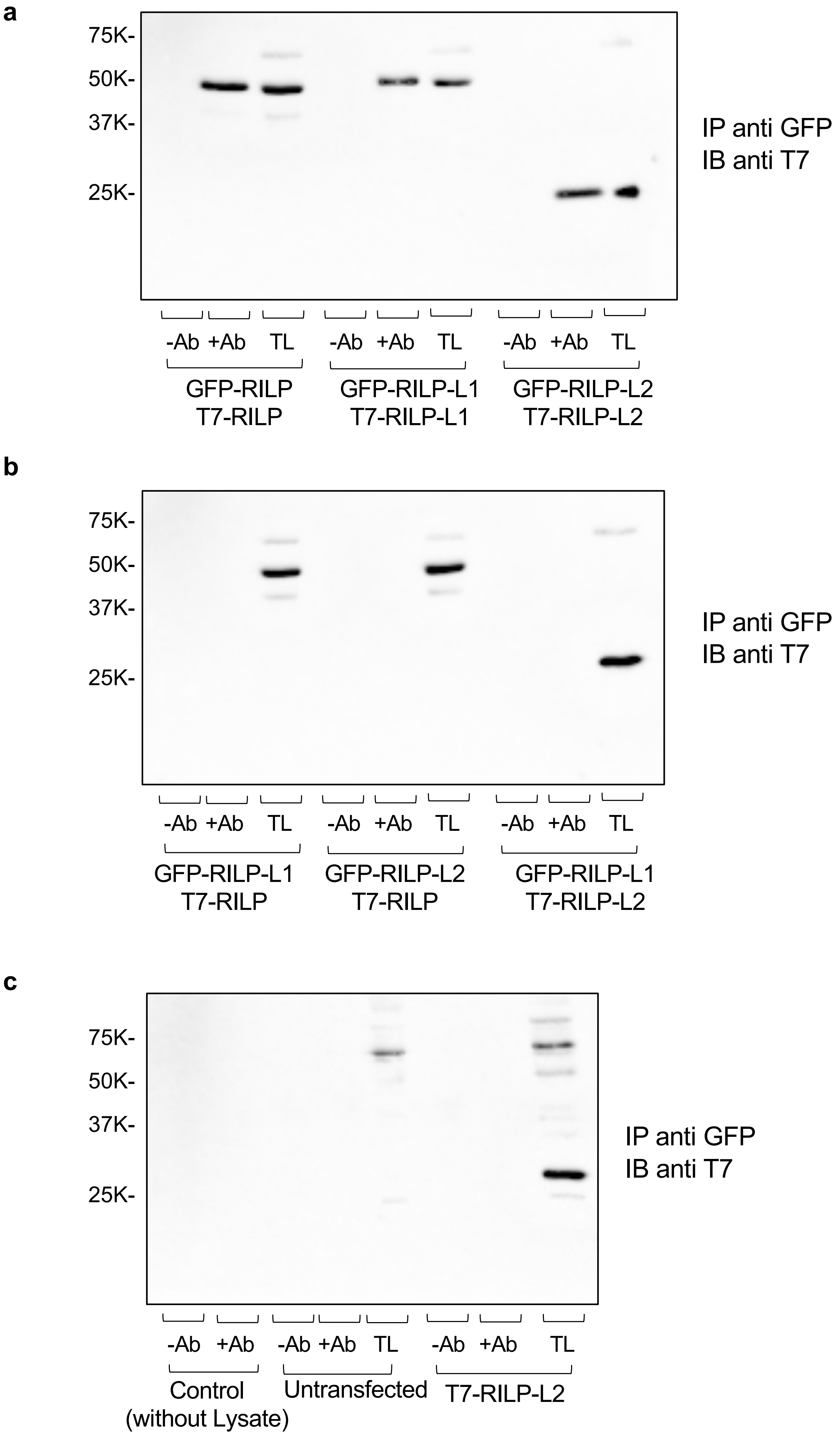
a
75K-
50K-
37K-
25K-
IP anti GFP
IB anti T7
-Ab +Ab TL -Ab +Ab TL -Ab +Ab TL
GFP-RILP
T7-RILP
GFP-RILP-L1
T7-RILP-L1
GFP-RILP-L2
T7-RILP-L2
b
75K-
50K-
37K-
25K-
IP anti GFP
IB anti T7
-Ab +Ab TL -Ab +Ab TL -Ab +Ab TL
GFP-RILP-L1
T7-RILP
GFP-RILP-L2
T7-RILP
GFP-RILP-L1
T7-RILP-L2
c
75K-
50K-
37K-
25K-
IP anti GFP
IB anti T7
-Ab +Ab -Ab +Ab TL -Ab +Ab TL
Control
(without Lysate)
Untransfected
T7-RILP-L2

Fig. 1

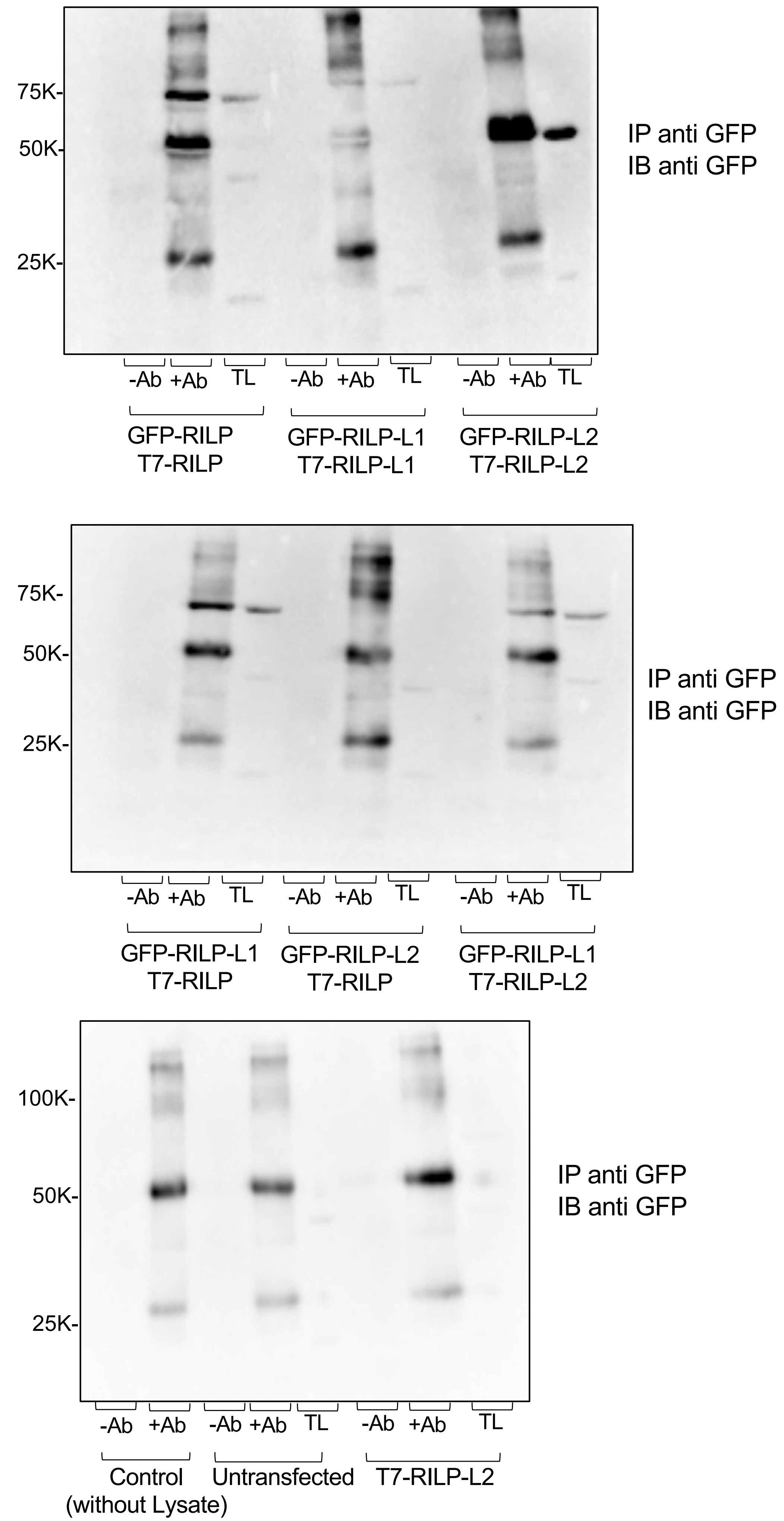
75K-
50K-
25K-
IP anti GFP
IB anti GFP
-Ab +Ab TL -Ab +Ab TL -Ab +Ab TL
GFP-RILP
T7-RILP
GFP-RILP-L1
T7-RILP-L1
GFP-RILP-L2
T7-RILP-L2
75K-
50K-
25K-
IP anti GFP
IB anti GFP
-Ab +Ab TL -Ab +Ab TL -Ab +Ab TL
GFP-RILP-L1
T7-RILP
GFP-RILP-L2
T7-RILP
GFP-RILP-L1
T7-RILP-L2
100K-
50K-
25K-
IP anti GFP
IB anti GFP
-Ab +Ab -Ab +Ab TL -Ab +Ab TL
Control
(without Lysate)
Untransfected
T7-RILP-L2

Fig. 1

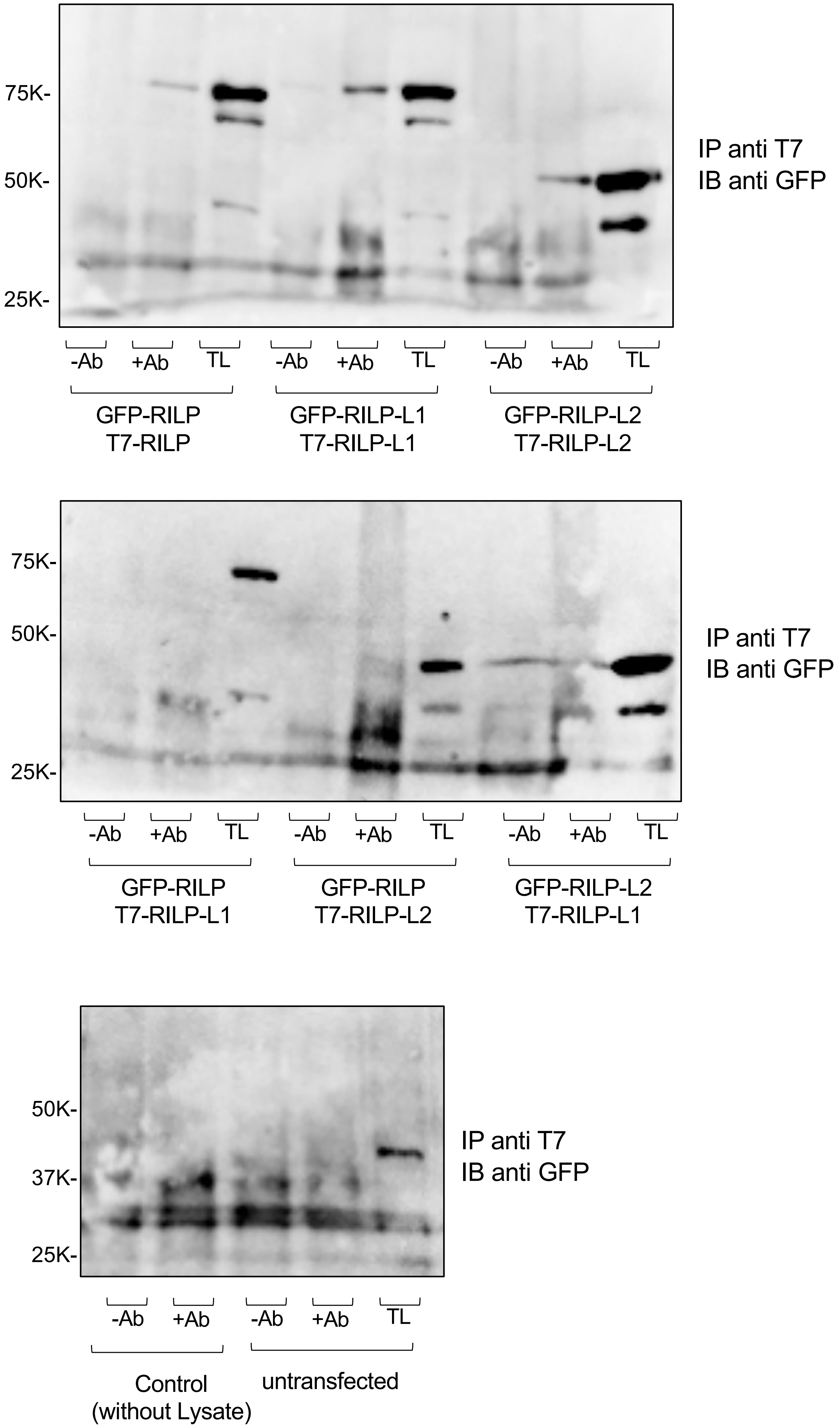
75K-
50K-
25K-
IP anti T7
IB anti GFP
-Ab +Ab TL -Ab +Ab TL -Ab +Ab TL
GFP-RILP
T7-RILP
GFP-RILP-L1
T7-RILP-L1
GFP-RILP-L2
T7-RILP-L2
75K-
50K-
25K-
IP anti T7
IB anti GFP
-Ab +Ab TL -Ab +Ab TL -Ab +Ab TL
GFP-RILP
T7-RILP-L1
GFP-RILP
T7-RILP-L2
GFP-RILP-L2
T7-RILP-L1
50K-
37K-
25K-
IP anti T7
IB anti GFP
-Ab +Ab -Ab +Ab TL
Control
(without Lysate)
untransfected

Fig. 1

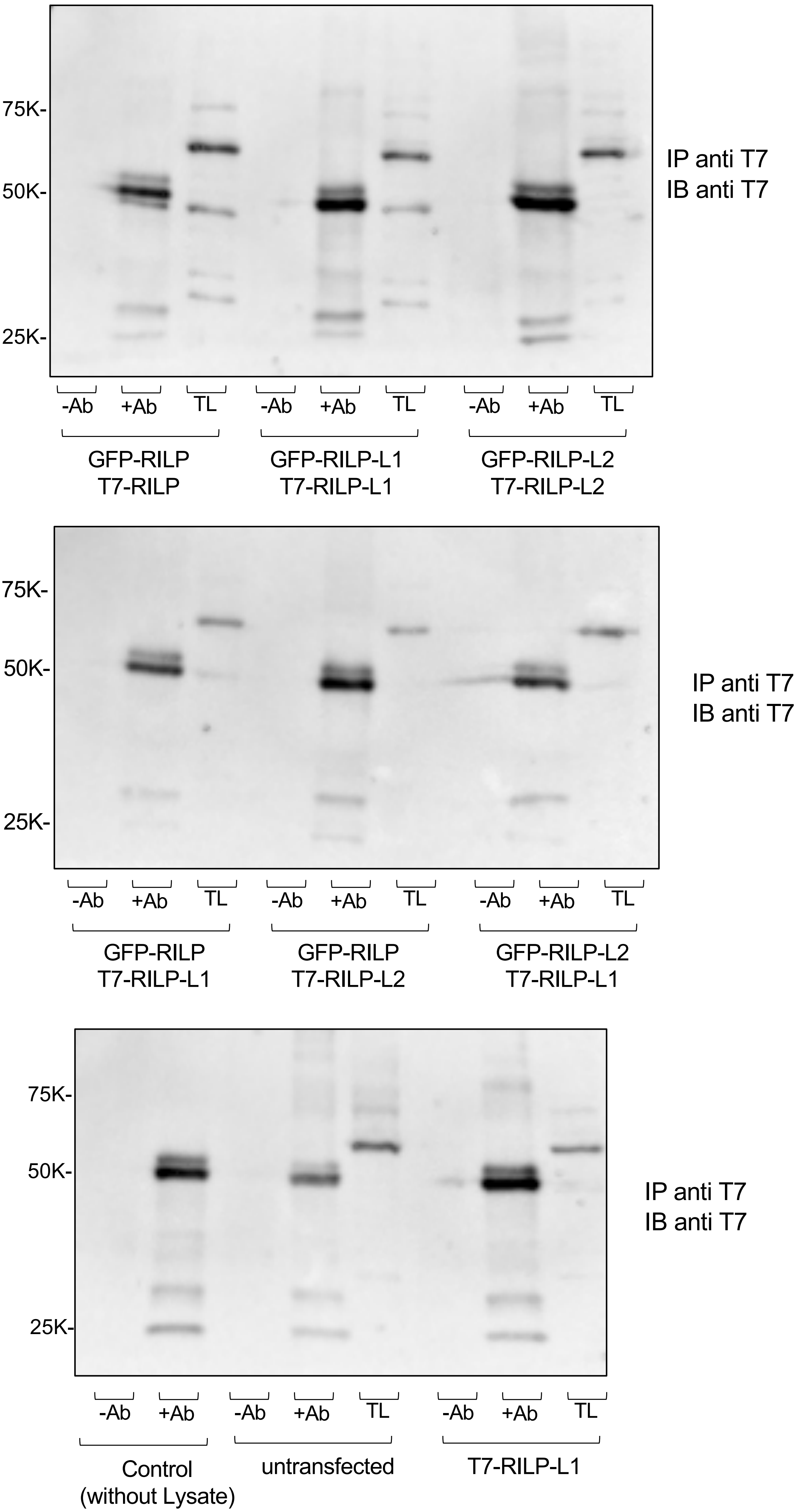
75K-
50K-
25K-
IP anti T7
IB anti T7
-Ab +Ab TL -Ab +Ab TL -Ab +Ab TL
GFP-RILP
T7-RILP
GFP-RILP-L1
T7-RILP-L1
GFP-RILP-L2
T7-RILP-L2
75K-
50K-
25K-
IP anti T7
IB anti T7
-Ab +Ab TL -Ab +Ab TL -Ab +Ab TL
GFP-RILP
T7-RILP-L1
GFP-RILP
T7-RILP-L2
GFP-RILP-L2
T7-RILP-L1
75K-
50K-
25K-
IP anti T7
IB anti T7
-Ab +Ab -Ab +Ab TL -Ab +Ab TL
Control
(without Lysate)
untransfected
T7-RILP-L1

Fig. 1

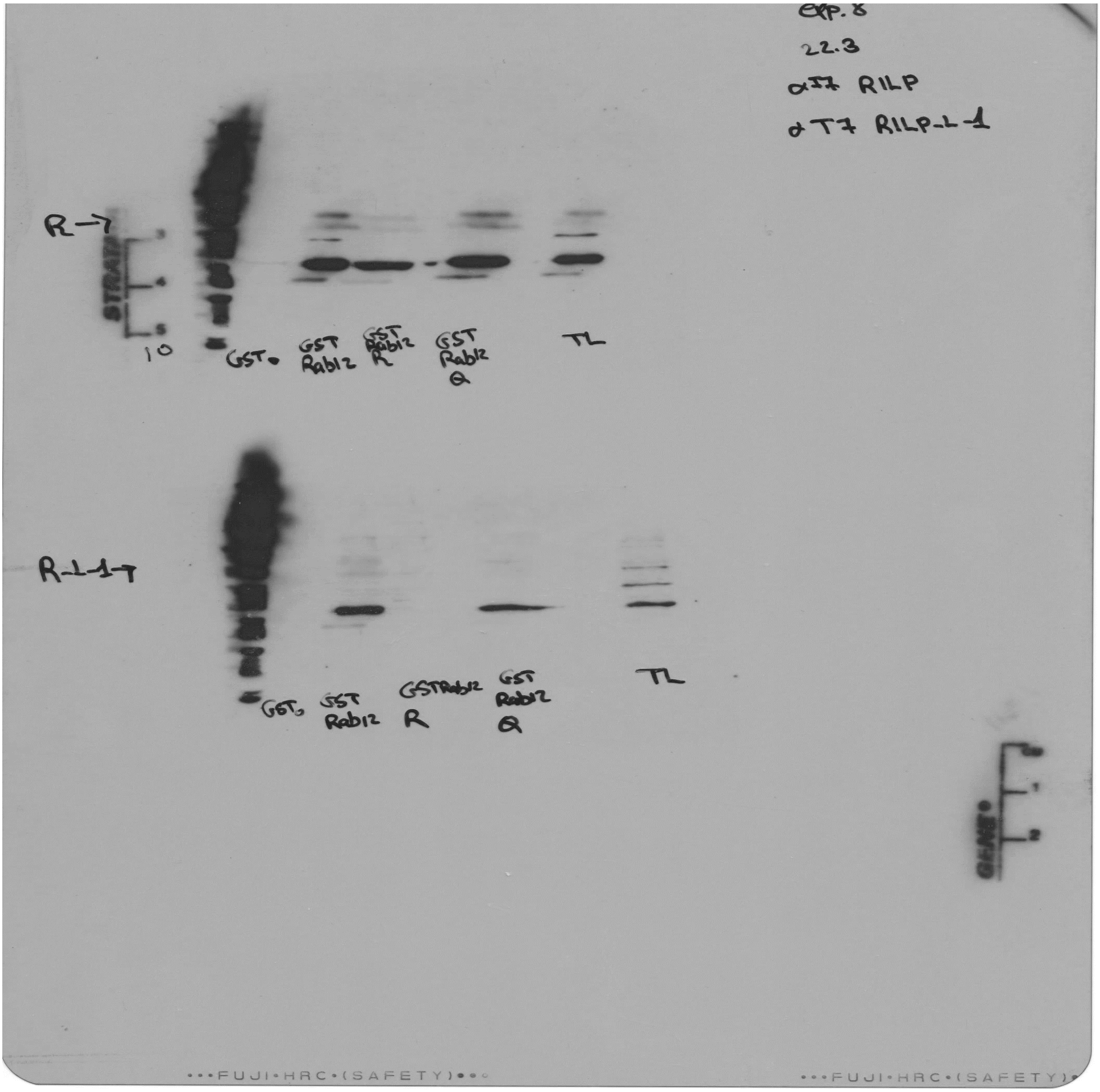
Exp. 8
22.3
αT7 RILP
αT7 RILP-L-1
R→
10
GST
GST Rab12
GST Rab12 R
GST Rab12 Q
TL
R-L-1→
GST
GST Rab12
GSTRab12 R
GST Rab12 Q
TL
•••FUJI•HRC•(SAFETY)•••
•••FUJI•HRC•(SAFETY)•

Fig. 2

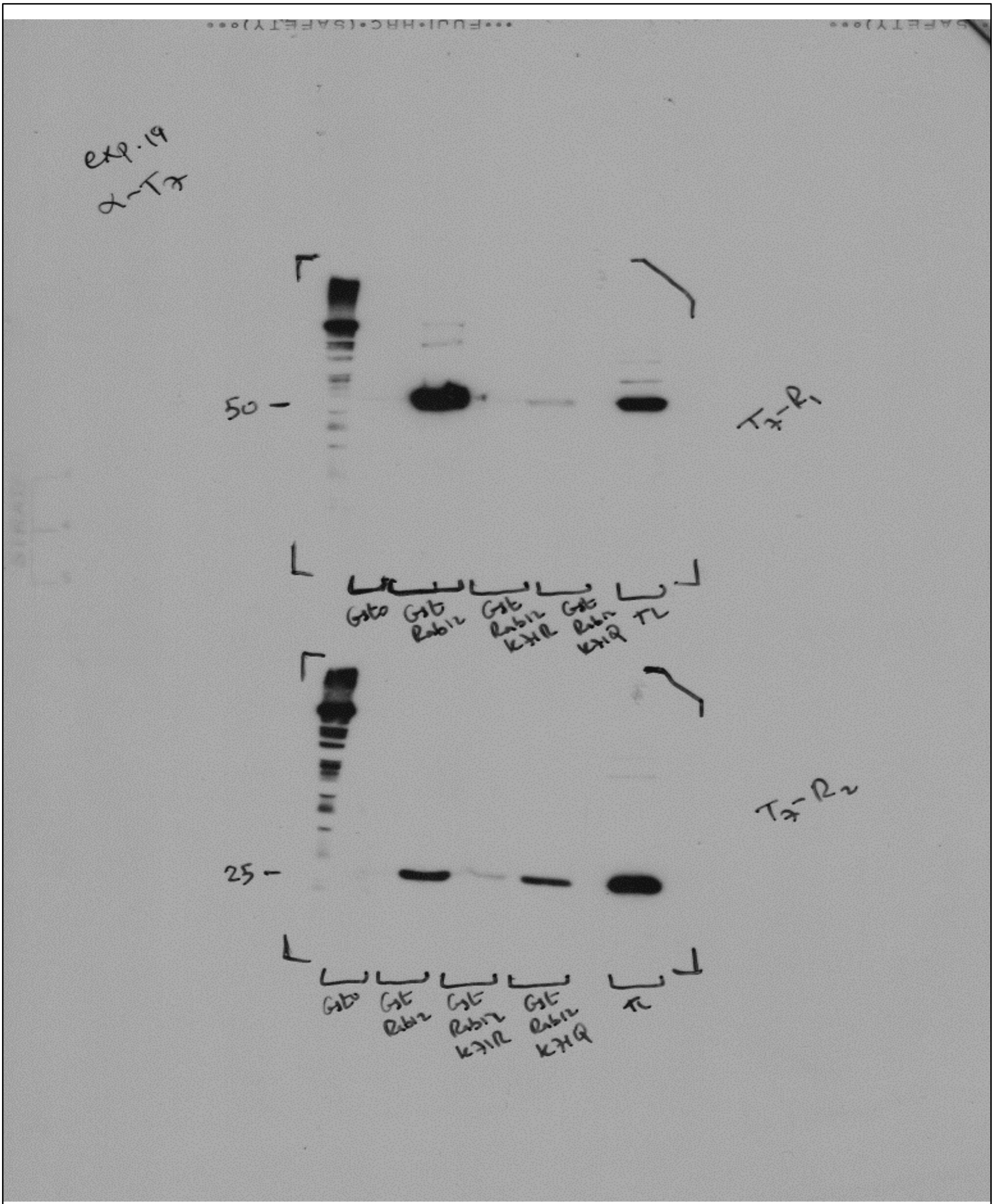

Fig. 2

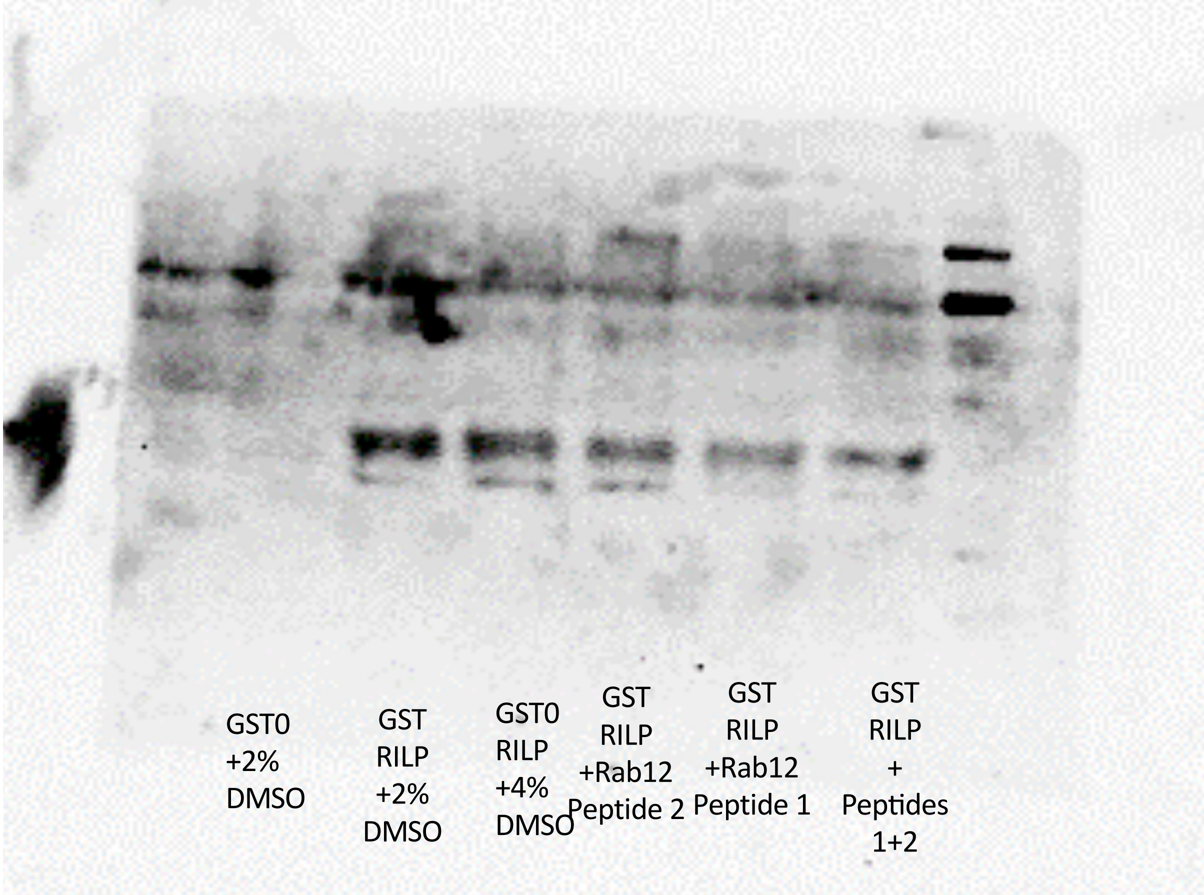
GST0 +2% DMSO
GST RILP +2% DMSO
GST0 RILP +4% DMSO
GST RILP +Rab12 Peptide 2
GST RILP +Rab12 Peptide 1
GST RILP + Peptides 1+2

Fig. 6

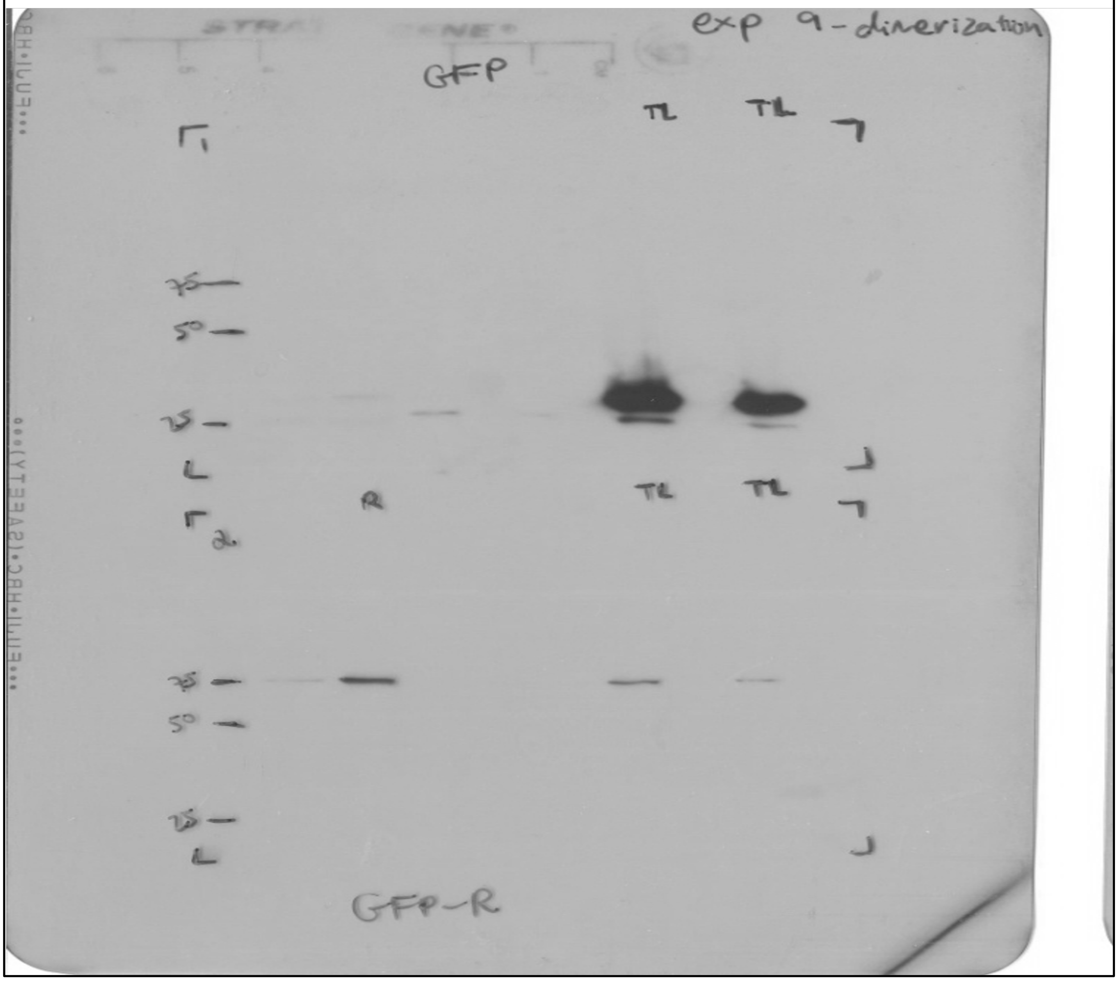

GST GST-RILP GST-RILP-L1 GST-RILP-L2 TL

Fig. S2

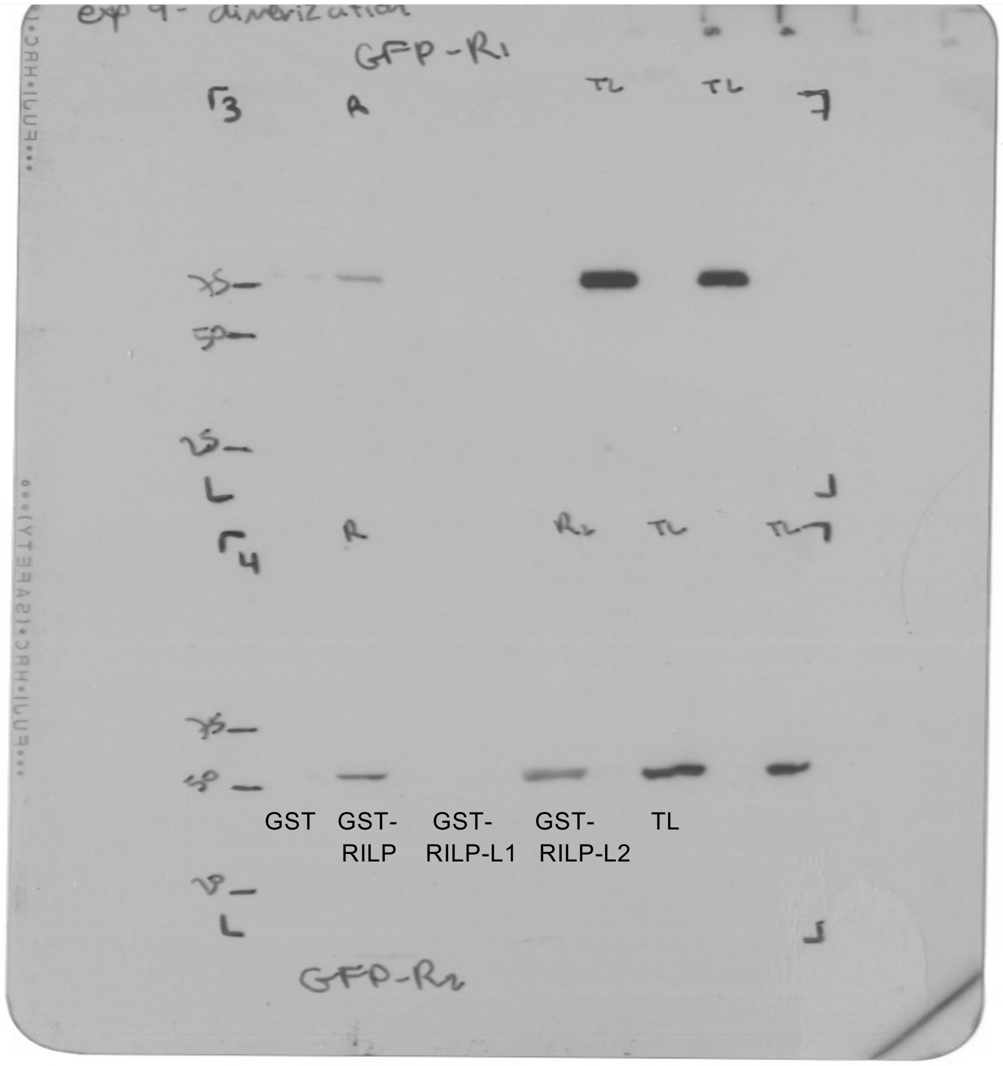
GFP-R1
TL
TL
R
R
R2
TL
TL
GST
GST-
RILP
GST-
RILP-L1
GST-
RILP-L2
TL
GFP-R2

Fig. S2

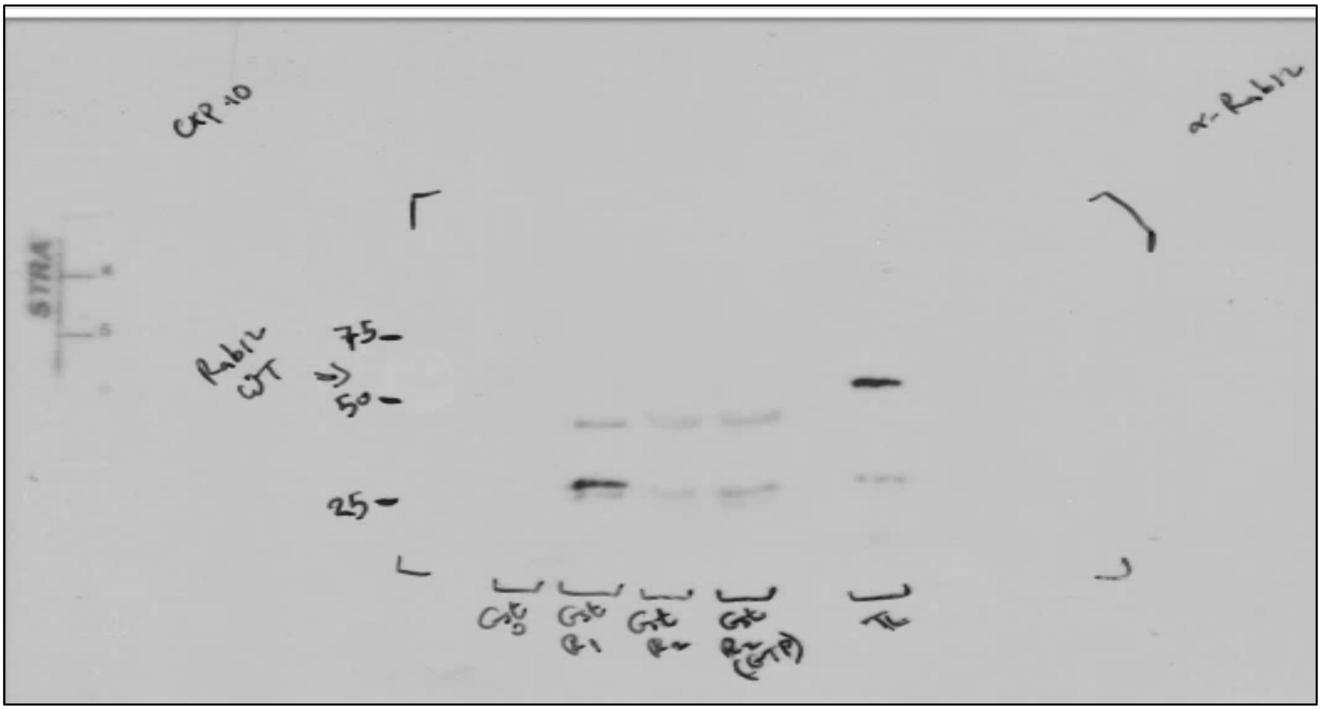

Fig. S2

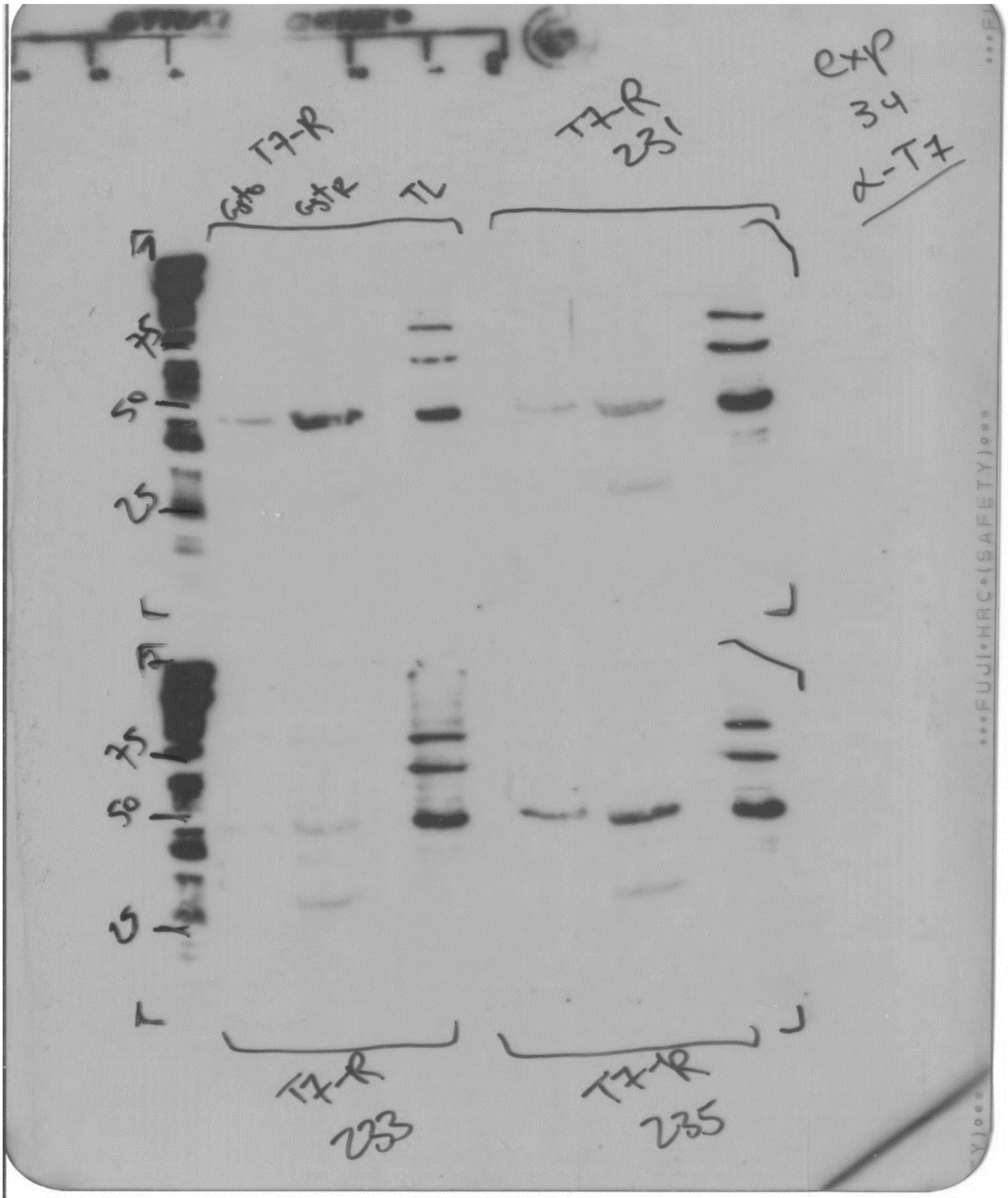

Fig. S3

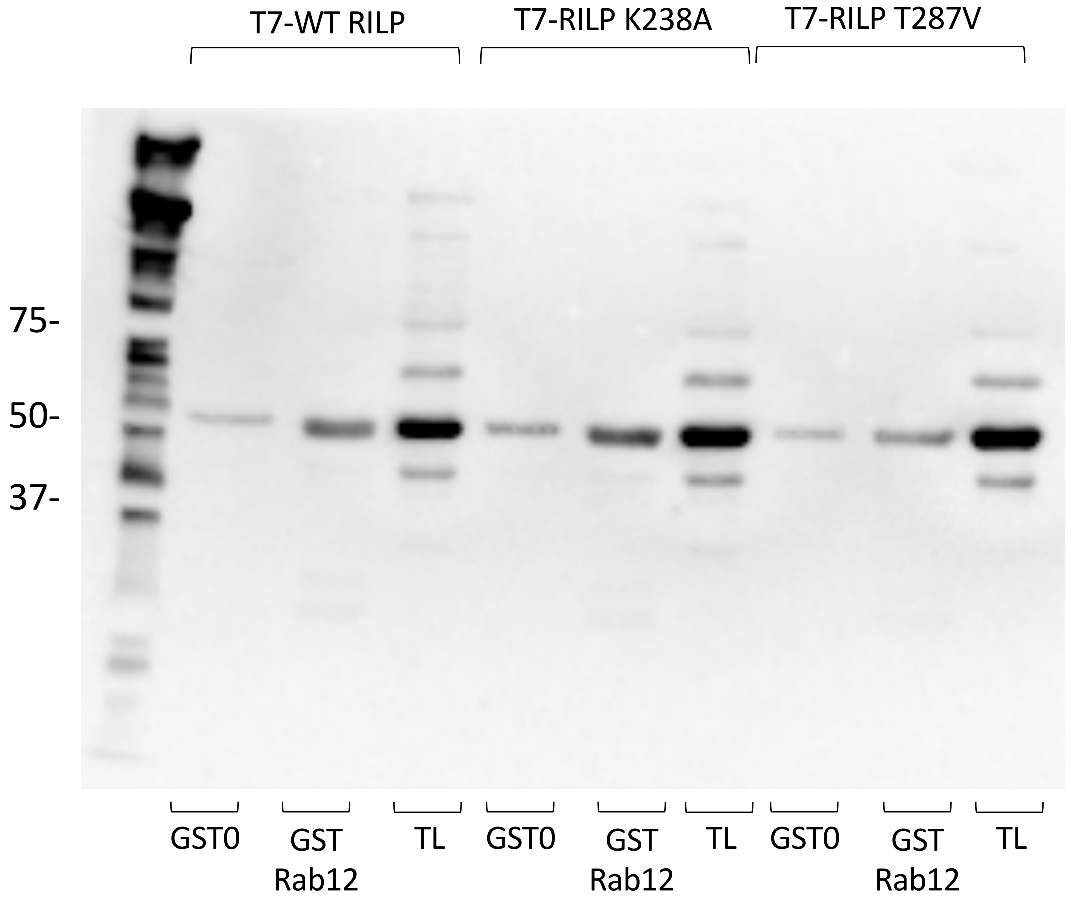
T7-WT RILP
T7-RILP K238A
T7-RILP T287V
75-
50-
37-
GST0
GST
Rab12
TL
GST0
GST
Rab12
TL
GST0
GST
Rab12
TL

Fig. S3
